# Metaproteomic profiling reveals viral proteins and associated host proteomic alterations in glioblastoma

**DOI:** 10.1038/s41598-026-58378-1

**Published:** 2026-06-22

**Authors:** Bavani Gunasegaran, Shivani Krishnamurthy, Seong Beom Ahn, Benjamin Heng

**Affiliations:** 1 Program in Translational and Clinical Liver Cancer Research, Division of Medical Science, National Cancer Centre Singapore, Singapore, Singapore; 2 Macquarie Medical School, Faculty of Medicine, Health and Human Sciences, Macquarie University, Sydney, NSW Australia; 3 School of Medical Sciences, Faculty of Medicine and Health, University of Sydney, Sydney, NSW Australia

**Keywords:** Viruses, Glioblastoma, Metaproteomic, Proteomics, Virome, Mass spectrometry, Herpes simplex virus, Biomarkers, Cancer, Computational biology and bioinformatics, Oncology

## Abstract

Glioblastoma (GB) is a WHO grade 4 brain cancer with dismal prognosis, yet its aetiology remains poorly defined. Although viral involvement has been proposed, findings across studies remain inconsistent, reflecting inherent limitations of individual technologies and cohort size. Here we applied metaproteomic profiling to a publicly available GB proteome dataset (12 control, 21 adjacent, 159 tumour) and an independent cohort of 81 samples (37 control, 44 tumour) to detect viral proteins in tumour and controls tissues. Across cohorts, we detected viral proteins from diverse species, with human herpesviruses (HHV-1, 2, and 8) more frequently detected in GB tumours compared with control tissues. Analysis of the host tumour proteome revealed differential abundance of proteins related to transcriptional regulation, RNA processing, protein translation, immune responses, and mitochondrial-associated metabolism. Correlation analysis identified associations between viral and human proteins, with several linked to biological processes previously implicated in DNA virus-host interactions. Further stratification of tumour by HHV-1 status showed consistent alterations in proteins associated with mitochondrial-associated metabolism, protein turnover, and cell adhesion/signalling.In summary, this study demonstrates the feasibility of metaproteomics for detecting viral components in archival GB tissues. Using this approach, we observed differences in viral protein landscape across cohorts and identified associations between viral presence and host proteomic features, providing a protein-level framework for future studies of virus-host interactions in GB.

**Supplementary Information:**

The online version contains supplementary material available at https://doi.org/10.1038/s41598-026-58378-1.

## Introduction

Glioblastoma (GB) is classified as a grade 4, isocitrate dehydrogenase (IDH)-wildtype^1^ brain tumour in adults^2^. It is characterised by hallmark genetic alterations including telomerase reverse transcriptase (TERT) promoter mutations, epidermal growth factor receptor (EGFR) amplification, and chromosomal gains and losses (+7/−10)^3,4^. Despite multimodal treatment encompassing maximal safe resection, radiation therapy, and temozolomide chemotherapy prognosis remains poor^5^, with a median survival of approximately 15 months and a five-year survival rate below 10%^6–8^. This can be attributed to the heterogeneity of the tumour and its microenvironment. As the aetiology of GB remains unclear, contributors beyond known genetic alterations such as environmental and biological factors have been explored, including the potential involvement of viruses. Interest in this area stems from well-established model of viral oncogenesis, such as human papillomavirus in cervical cancer^9–11^, raising the possibility that viral elements may influence GB biology.

To date, more than 63 studies have explored the presence of viruses in GB, utilising a wide spectrum of technologies ranging from polymerase chain reaction to next-generation sequencing (NGS) technologies^12^. These studies have progressively advanced our understanding potential viral associations^12^, reporting viruses such as Epstein-Barr virus, polyomaviruses and retroviruses, with human cytomegalovirus being the most frequently detected^13,14^. Although these findings are important, its prevalence and causal role remain inconsistent across studies, largely due to inherent limitations unique to each technology. This thus highlight the complexity of investigating virome within human tumours.

While genetic evidence remains fundamental to understanding viral presence, assessing viral protein expression is also essential for understanding potential biological role^15,16^. Metaproteomics has emerged as a complementary approach that enables the simultaneous profiling of human and viral proteome. Its increasing use in cancer research (47 results on PubMed when searched for metaproteomics and virus, assessed on 23^rd^ March 2025), reflects its capacity to study host-microbe interactions and potentially, the functional microbial activity^16–19^. Unlike conventional targeted molecular assays restricted to pre-defined viral candidates^12^, this approach leverages unbiased mass spectrometry-based metaproteomics and bulk proteomics to detect viral peptides. Furthermore, it also addresses a key challenge in virome studies: The absence of a universal genetic marker, in contrast to bacterial 16S ribosomal RNA, making proteomic evidence particularly informative for evaluating viral presence and activity.

In this study, an integrative metaproteomic approach was applied to detect viral proteins in GB tumours and examine associated host proteomic alterations using a publicly available proteome database and an Australian cancer biobank cohort. Across both cohorts, human herpesviruses (HHV) were observed to be the predominantly present in GB tumours. Leveraging this approach, we identified dysregulation of immune, metabolic, and structural networks within the human tumour proteome, with these alterations being enriched in HHV-1 positive tumours. Lastly, correlation analyses further revealed associations between viral proteins and host cytoskeletal, metabolic, and ribosomal functions. Together, these findings revealed viral-associated proteomic signatures in GB and provide a foundation for exploring how persistent viral presence may influence tumour biology.

## Methods

### Study samples

Archived FFPE tissue samples were acquired from a retrospective cohort of 48 GB tumour and 48 control brain tissues from the NSW Regional Biospecimen Services, formerly known as Hunter Cancer Biobank (HCB), Newcastle, New South Wales, Australia. These samples were obtained post-mortem during autopsy procedures.

The HCB cohort comprised of 48 patients (20 females and 28 males) with a mean age of 63.5 years, while the control cohort included 48 individuals (17 females and 31 males) with a mean age of 62 years. Inclusion criteria required a confirmed diagnosis of GB as the primary cancer and IDH-negative status by IHC. Our study was approved by the Macquarie University Human Research Ethics committee (Ref. 520251101062307). All research methods were performed in accordance with the relevant guidelines and regulations, and informed consent was obtained from all participants and/or their legal guardians.

### Public meta-proteome data collection

The study utilised the publicly available GB datasets from the ProteomeXchange Consortium with the identifier PXD038732^20^ (accessed on 1^st^ February 2024), generated by Fudan University (FDU). These FDU cohort comprised data-dependent acquisition (DDA) raw mass spectrometry (MS) files, including 12 control brain tissues, 21 tumour-adjacent tissues, and 159 GB tumour samples. Detailed cohort demographics and gender information for all samples from both cohorts are provided in the Supplementary Materials (Supplementary File 1).

### Recombinant proteins

The following recombinant proteins were used to construct the DDA recombinant protein spectral library for this study: AADAT (R&D Systems, 7927-AT-010), AFMID (OriGene Technologies, TP317126), BCL2 (OriGene, TP304498), C1QC (OriGene, TP761200), CDX2 (Aviva Systems Biology, OPCD02118), CEACAM5 (R&D Systems, 4128-CM), CXCL10/IP10 (OriGene, TP723726), CXCR3 (Abnova, H00002833-P01), CYP1A1 (OriGene, TP305760), CYP1A2 (OriGene, TP321636), CYP1B1 (Abcam, AB114353), EGF (MyBioSource, MBS650012), EGFR (OriGene, TP314877), ELF3 (Abnova, H00001999-P01), FOXP3 (Aviva, OPCD03302), IDO1 (R&D Systems, 6030-AO-010), IDO2 (R&D Systems, 9967-AO-050), KMO (R&D Systems, 8050-KM-025), KPNA2 (Aviva, OPCD04722), KRT17 (Aviva, OPCD04744), KRT20 (OriGene, TP760147), KYNU (R&D Systems, 4887-KH-010), MIA (R&D Systems, 9250-1A), MMP9 (R&D Systems, 911-MP), MUC1 (OriGene, TP760771), MUC16 (Aviva, OPCD01793), MYC (OriGene, TP301611), PDGFB (OriGene, TP723355), PFN1 (Novus Biologicals, NBP1-30215), PGCP (OriGene, TP760108), PTEN (Novus, 847-PN-010), QPRT (OriGene, TP302960), S100A8/S100A9 (Novus, 8226-S8), SATB1 (Abnova, H00006304-P01), TDO2 (R&D Systems, 9768-TD-020), TGFA (OriGene, TP723858), TGFB1 (OriGene, TP300973), TIMP1 (R&D Systems, 970-TM), TNFα (R&D Systems, 2100-TA-005/CF), sTNF-R1/TNFRSF1A (OriGene, TP723870), TP53 (Novus, SP-454), TYMS (Aviva, OPCD07780), and VEGF165 (R&D Systems, 293-VE-010/CF).

### Protein extraction from FFPE tissue

To remove the paraffin, 1 mL of xylene was added to each tube of sample, agitated for 5 minutes, and then discarded by centrifugation at 14,000×g for 5 minutes. This step was followed by the addition of 1 mL of 100% ethanol, agitation for 5 minutes, and centrifugation at 14,000×g for 5 minutes to discard the ethanol. The tissue pellet was briefly air-dried before adding 160 µL of 0.1 M triethylammonium bicarbonate buffer (Sigma-Aldrich, MO, USA) and sonication (Branson Sonifier 450) at an output setting of 2 with an amplitude of 40% for 30 seconds. Sodium deoxycholate (Sigma-Aldrich, MO, USA) was then added to a final concentration of 2% w/v, and the samples were incubated at 99°C for 60 minutes. Reduction was carried out with Dithiothreitol (Sigma-Aldrich, MO, USA) at a final concentration of 25 mM, incubating at 56°C for 30 minutes, followed by alkylation with Iodoacetamide (Sigma-Aldrich, MO, USA) at a final concentration of 75 mM, incubating at room temperature for 30 minutes in the dark. For acetone precipitation, the sample was transferred to a 1.5 mL Eppendorf tube, followed by adding a 4× volume of ice-cold acetone and freezing overnight at −20°C. The sample was then centrifuged at 20,800×g at 4°C for 30 minutes. The supernatant was carefully removed and discarded, ensuring not to disturb the pellet. The pellet was washed twice with 500 µL of ice-cold 80% (vol/vol) acetone, each followed by centrifugation at 20,800×g at 4°C (10 minutes for the first wash and 2 minutes for the second wash). The protein pellet was air-dried by keeping the tube open for 5 minutes. The pellet was resuspended in 100 µL of 0.1 M triethylammonium bicarbonate buffer and sonicated (output setting of 2, amplitude of 40%), and protein concentration was measured using Pierce^TM^ BCA protein assay kit (Thermo Fisher Scientific, MA, USA) according to the manufacturing protocol. All protein samples were normalised to the same concentration, and digested with sequencing grade porcine trypsin (Promega, WI, USA) at a protease to substrate ratio of 1:30 at 37 °C for 16 h at 600 rpm.

### Recombinant protein spectral library

A recombinant protein spectral library was generated as previously described^21^ by pooling the selected recombinant proteins. The proteins were treated with 15 mM dithiothreitol for 30 minutes at 60 °C, to reduce disulfide bonds, followed by alkylation with 30 mM iodoacetamide in the dark for another 30 minutes at room temperature. Proteins were digested with trypsin at a 1:20 enzyme-to-substrate ratio for 16 hours at 37 °C with gentle agitation. The resulting peptides were purified using C18 StageTips method^22^.

### Sample preparation for tandem mass spectrometry

10% formic acid was added to achieve a final concentration of 1% to precipitate any remaining sodium deoxycholate. The precipitate was spun out at 14,000×g for 5 minutes, the supernatant was transferred to a new tube, and the precipitation step was repeated. Finally, the samples were dried using vacuum centrifugation for approximately 90 min. Samples were subsequently desalted and cleaned with C18 StageTips method^22^.

### LC-MS/MS analysis

The samples were obtained using two approaches: DDA for analysing viral peptides and Data Independent Acquisition (DIA) for analysing human peptides, in individual samples. DDA was also employed to construct a library for DIA file analyses. A Vanquish ultra-high-performance liquid chromatographer (UHPLC) system paired with an Orbitrap Exploris 480 mass spectrometer from Thermo Fisher Scientific (MA, USA). For each run, 600 ng of peptides were loaded onto a trap column and washed with 0.1% formic acid in water (loading buffer). The trap column was then switched in-line with a custom-packed analytical nano-LC column (ReproSil-Pur 120 C18-AQ, 3 µm, 250 × 0.075 mm; 75 µm × 30 cm).

Peptide separation was performed using a linear gradient of mobile phase B, increasing from 2.5% to 37.5% over 60 minutes for DIA and 86 minutes for DDA, at a flow rate of 300 nL/min. This was followed by a hold at 95% mobile phase B for 10 minutes in DIA and 7 minutes in DDA runs. The eluted peptides were introduced into the mass spectrometer operating in positive ion mode. Twenty high-pH fractions were analysed using the DDA method for library construction, while individual samples intended for quantitation were analysed using the DIA method, as detailed below.

## Proteomic analysis

### Proteome DDA and Protein spectral library generation

A protein spectral library was created using the above 20 fractionated peptides, which were developed independently. The column eluent was introduced into the mass spectrometer’s ionisation source, which operated in positive ion mode. Survey scans ranging from 350 to 1450 m/z were recorded at 60 K resolution, specifically at 200 m/z, utilizing the orbitrap analyser. Precursor ions, with a minimum intensity of 5 × 10^3^ and charge states ranging from +2 to +6, underwent MS/MS analysis with a fixed cycle time of 1.5 seconds. The precursor survey scans were fragmented by higher-energy collisional dissociation (HCD) at a normalised collision energy of 27, and at a resolution of 15 K at 200 m/z. Dynamic exclusion was activated, with a 15-second exclusion duration and a tolerance of ± 10 ppm.

### Proteome DIA

The column eluent was directed into the ionisation source of the mass spectrometer, which was set to operate in positive ion mode. MS1 scan was conducted over a range of 350 to 1,450 m/z at a resolution of 60 K. This was followed by MS2 scans at 20 m/z intervals, which were fragmented using HCD with a normalised collision energy of 27. The scan resolution for the MS2 scans was set at 30 K. The parameters for Automatic Gain Control were configured to “Standard” and “Auto" for maximum injection time.

### Data processing for DDA spectral library and DIA proteome

High pH library fractions DDA data was utilised to generate an ion library using MSFragger (version 4.1), Python (version 3.9.13), and EasyPQP (version 0.1.50) in the Fragpipe GUI (version 22.0)^23^. DDA files were searched against a database of human proteins (reviewed Uniprot proteome UP000005640, downloaded on December 2024 containing 20,421 protein sequences with reversed sequences included as decoy targets) in the default workflow. Trypsin was specified as the digestion enzyme, with a peptide length range of 9-30 amino acids, a peptide mass range of 500-5000 Da, and a maximum of two missed cleavages. Precursor and fragment mass tolerances were both set to 20 ppm. Methionine oxidation and N-terminal acetylation were included as variable modifications, whereas cysteine carbamidomethylation was defined as a fixed modification. A maximum of three variable modifications per peptide was allowed. Following the search, a spectral library of b- and y-type fragment ions was generated using SpecLib. Retention time calibration was performed with automatic selection, using RT Lowess smoothing with a fraction value of 0.01. The Unimod tolerance was set to 0.02 Da, and the fragment ion tolerance was set to 15 ppm.

### DIANN data analysis and statistics

Protein quantification was performed using DIA-NN (version 1.9.2), based on the spectral library constructed as described above. A double-pass neural network classifier was applied for identification and quantification^24^. MS1 accuracy, mass accuracy, and scan window width were all set to 0, allowing automatic parameter optimisation. The "match between runs" feature was enabled to improve quantification consistency across samples. Precursor-level false discovery rate (FDR) was controlled at 1%. Protein inference was conducted at the protein name level based on the FASTA database. Quantification was performed using the ‘quant UMS (high precision)’ strategy, and retention time (RT)-dependent cross-run normalisation was applied.

### Viral reference proteomes

Viral reference proteins (Unreviewed and reviewed) and human proteins (Reviewed) were downloaded from UniProt (downloaded December 2024, containing 148,5439 protein sequences with all sequences reversed as decoy targets) in FASTA format as described by He et al. 2022^25^.

### Trans-proteomic pipeline analysis and statistics

Trans-Proteomic Pipeline (TPP) (version 7.1.0) was utilized for the analysis of the metaproteomic MS datasets^26^. Raw Thermo MS files were converted to mzML format using TPP for Comet peptide search. PeptideProphet was then employed to validate the Comet search results for Peptide-Spectrum Match (PSM) validation. iProphet was subsequently applied within TPP to integrate and refine PSM probabilities across all samples by leveraging inter-run peptide identification consistency, providing an additional layer of validation beyond individual PSM-level scoring. Protein-level inference was then performed using ProteinProphet. All of the above was performed as described by He et al^25^. Comet searches were performed using default parameters, with a peptide mass tolerance set at 10 ppm. Peptide validation was carried out using PeptideProphet within TPP. Accurate mass binning was applied in ppm, with a minimum peptide length threshold of 9 amino acids. Negative distribution for score modelling was defined by utilising known decoy hits^25^.

Data retrieved from ProteinProphet were processed in R (version 4.4.2) using RStudio (version 2024.09.1, build 394). To ensure high quality of data, protein identifications with low-probability assignments according to ProteinProphet were excluded to maintain a 1% FDR at the protein level. Proteins of human origin and those without clear assignment to one species were filtered out in an additional step. Only proteins identified by at least one unique peptide (with a minimum length of nine amino acids) and detected in at least two samples were retained for downstream analysis. In addition, only viral species represented by two or more unique proteins were included in subsequent analyses.

The identified species were cross-referenced with the NCBI taxonomy database (https://ftp.ncbi.nlm.nih.gov/pub/taxonomy/new_taxdump; accessed on 3 February 2025), and the taxonomic data were visualized as sunburst plots using the ‘plotly’ package^27^.

### Ingenuity pathway analysis (IPA) analysis

IPA (Ingenuity Systems/Qiagen, Redwood City, CA, USA) was utilised to associate significant protein lists (FDR ≤ 0.05) with biological pathways. Canonical and functional pathway analyses were performed to identify relevant biological functions and pathways. Pathways were considered statistically significant if they met a z-score threshold of ±2 and a p-value < 0.05, as determined by Fisher’s exact test.

### Data visualisation and figure generation

Figures were generated using R packages. Explanatory figures were created using BioRender.com.

### Statistical analyses

The DIA host proteomics dataset (HCB cohort) and the DDA metaproteomic datasets (HCB and FDU cohorts) were analysed entirely independently using separate pipelines and statistical frameworks. The HCB and FDU metaproteomic datasets were treated as two independent experiments and are reported separately throughout; no results were pooled or jointly modelled across cohorts or acquisition methods.

*T* test and Pearson’s Chi-square test were used to analyse the difference of age and sex between the groups of HCB cohort respectively. Statistical analyses of host DIA proteomics data were performed using R. Raw DIA-NN peptide data was filtered to retain protein groups present in at least 70% of samples across the entire cohort (≥68 samples) to ensure data robustness. Normalisation was performed by aligning each sample’s median peak intensity to the global median. Missing data were handled using a Missing Not At Random (MNAR) imputation implemented in R (v4.4.2), with missing values replaced by values drawn from a Gaussian distribution shifted 3 standard deviations below the mean of observed log-transformed intensities (downshift = 3, width = 0.3), with a fixed random seed (set.seed(123)) for reproducibility^28–30^. For each variable, missing entries were stochastically imputed with values drawn from a distribution shifted below the mean of the observed intensities, with variance informed by the observed standard deviation. This strategy places imputed values toward the lower end of the intensity distribution, thereby approximating signals expected from low-abundance proteins^31^.

To evaluate protein abundance differences between groups, a t-test was conducted in the R statistical environment, with ANOVA p-values adjusted for multiple testing using the Benjamini-Hochberg method for FDR control. Differential expression between tumour and control brain tissues was defined by a combined threshold of statistical significance (p ≤ 0.05) and ±2-fold change, an approach previously validated in DIA spike-in studies for effective control of false discovery rates^30,32^.

To identify potential associations between viral and host proteins in GB samples, we performed correlation analysis using a custom R script (v4.2.0) implemented in RStudio. Viral protein detection was encoded as a binary variable (1 = detected in a given sample, 0 = not detected), reflecting true presence or absence of identification rather than a missing data substitution. Absence of detection represents a meaningful biological state and no imputation was applied. Point-biserial correlation was used to measure the association between this dichotomous variable and continuous human protein abundance across samples. For each viral-human protein pair, Pearson correlation coefficients and corresponding p-values were computed. Pairs with invariant viral data (e.g., all 0 or 1) were excluded. The resulting p-values were adjusted for multiple testing using the Benjamini-Hochberg method, and protein pairs with adjusted p ≤ 0.05 were retained as statistically significant.

## Results

### Identification of viral-associated peptides and proteins in public and biobank cohort

**Fig. 1:**
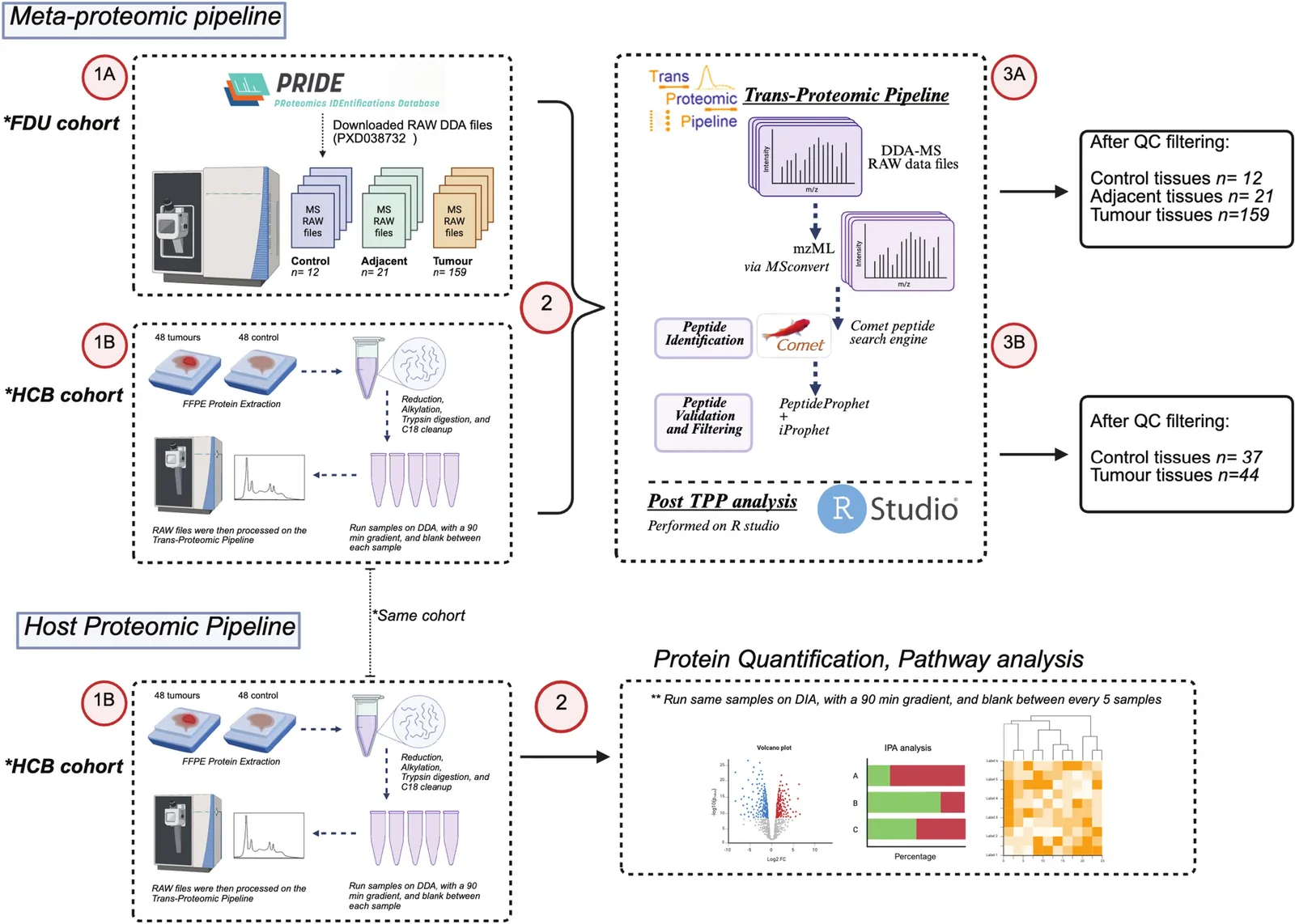
Workflow of metaproteomics and proteomics approach utilised for viral and human peptide/protein identification, validation, and quantification.Two study cohorts were used in this study: a publicly available GB dataset (PXD038732; Fudan University, FDU) and the Australian Cancer Biobank (Hunter Cancer Biobank; HCB). The FDU cohort consists of 12 control brain tissues, 21 tumour-adjacent tissues, and 159 GB tumour samples, while the HCB cohort includes 48 GB tumour and 48 control brain formalin-fixed, paraffin-embedded (FFPE) tissues. Data-dependent acquisition (DDA) was used for the analysis of viral peptides, while data-independent acquisition (DIA) was used for the analysis of human peptides in individual samples. The Trans-Proteomic Pipeline (TPP), Comet peptide search engine, and PeptideProphet were employed for the analysis of metaproteomic viral mass spectrometry datasets. DIA-NN and Ingenuity Pathway Analysis (IPA) were used to quantify human proteins and identify enriched pathways.

Viral proteins frequently mediate oncogenic activity in viral-associated cancers, underscoring the importance of profiling viral protein expression. To characterise viral protein presence in GB, a metaproteomic workflow was applied to analyse proteome data from two independent cohorts (Fig. 1). This included a publicly available dataset (FDU cohort), comprising 12 control brain tissues, 21 tumour-adjacent brain tissues, and 159 GB tumour tissues, as well as an Australian cancer biobank cohort (HCB cohort), comprising 48 control brain tissues and 48 GB tumour tissues. Across both cohorts, viral and host proteins were jointly assessed to enable integrated analysis of viral detection and corresponding host proteomic features. Clinical and molecular characteristics of the HCB and FDU cohorts are summarised in Table 1 and Supplementary File 1, respectively. While the publicly available FDU cohort, was analysed in its entirety, 15 samples were excluded from HCB cohort due to poor spectral signals, resulting in a final dataset of 81 samples (37 control brain and 44 GB tumour tissues) used for downstream metaproteomic analysis.

**Table 1:** HCB demography and characteristics: IDH1 = Isocitrate dehydrogenase; GFAP = Glial fibrillary acidic protein; ATRX = Alpha-thalassemia X-linked Intellectual disability; n = number.

	Normal controls (n=48)	GB (n=48)	*p-*value
Age in years (mean; range)	62 (48–72)	63.5 (55–72)	0.58
Sex (F; % female)	17 (35.4)	20 (41.7)	0.53
**Molecular and immunohistochemical features**			
IDH1 (%)			
Wildtype	NA	48 (100)	
Mutant	NA	0 (0)	
**GFAP (%)**			
Positive	NA	47 (97.9)	
Negative	NA	1 (2.1)	
**Ki67 index (%)**			
<10%	NA	0 (0)	
11–20%	NA	3 (6.3)	
21–30%	NA	8 (16.7)	
31–40%	NA	6 (12.5)	
>40 or high	NA	4 (8.3)	
Not reported	NA	27 (56.3)	
**p53 (%)**			
Mutant	NA	16 (33.3)	
Wildtype	NA	18 (37.5)	
Not reported	NA	14 (29.2)	
**ATRX (%)**			
Retained	NA	23 (47.9)	
Mutated	NA	6 (12.5)	
Nor reported	NA	19 (39.6)	
**Location of tissue**			
Frontal lobe/ Frontal cortex	19 (39.6)	NA	
Midbrain/ Brain stem	15 (31.3)	NA	
Parietal lobe	4 (8.3)	NA	
Occipital lobe	3 (6.3)	NA	
Temporal lobe	2 (4.2)	NA	
Cerebellum	1 (2.1)	NA	
Cerebellum (non-lobar)	1 (2.1)	NA	
Vascular territory (ACA/MCA)	2 (4.2)	NA	
Not recorded	1 (2.1)	NA	

**Fig. 2:**
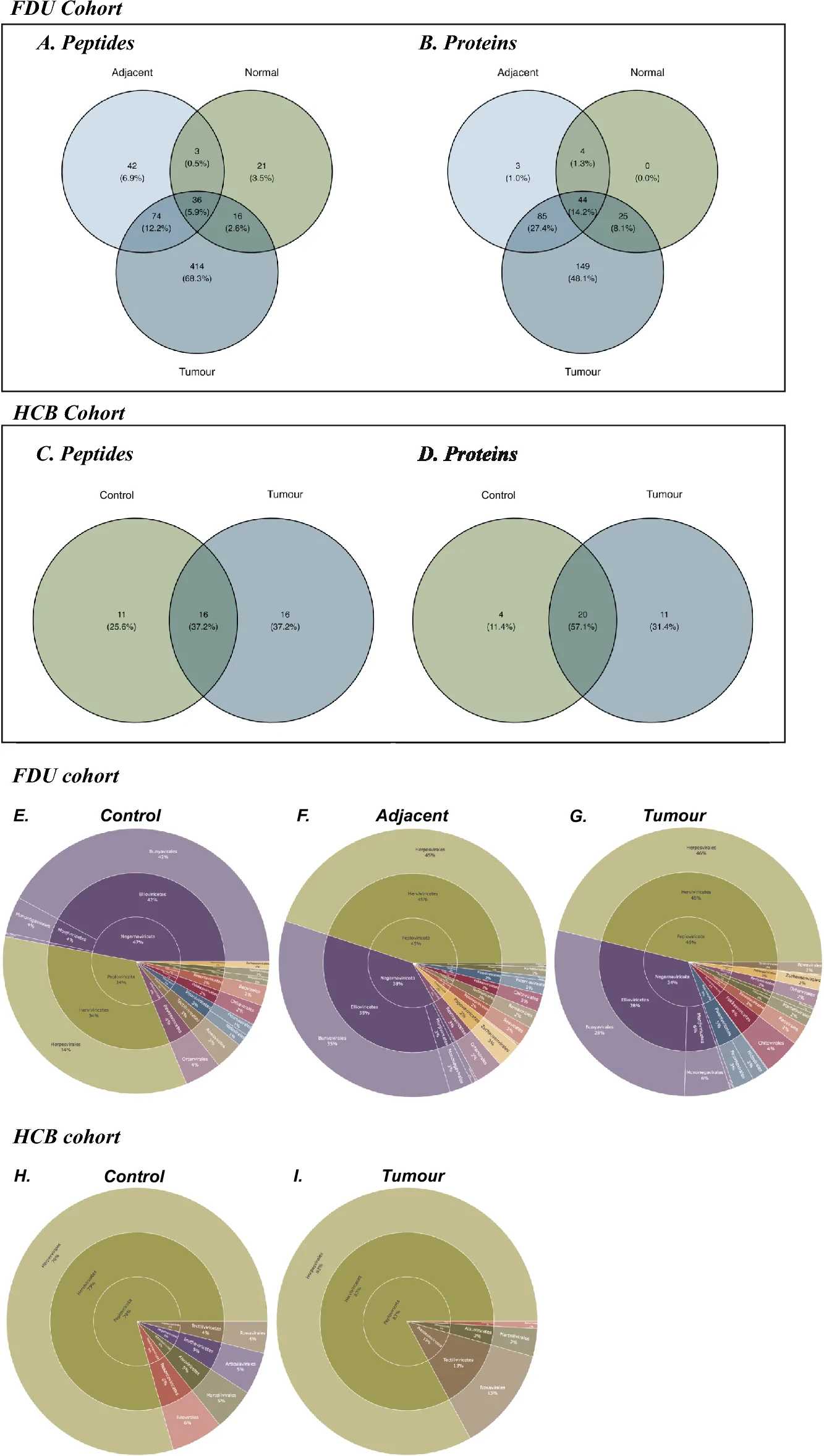
Venn diagrams displaying the distribution of viral peptides and proteins between tissue types in FDU and HCB cohort. Venn diagrams displaying the distribution of viral (**A**) peptides and (**B**) proteins between control, adjacent, and tumour samples in the FDU cohort, (**C**) peptides and (**D**) proteins between control and tumour samples in the HCB cohort, identified in at least 2 samples. Metaproteomic sunburst plots illustrating the hierarchical taxonomic composition of viruses in brain tissue samples from FDU and HCB cohort. The plots represent viral distributions in (**E**) control brain tissue, (**F**) adjacent tissue, and (**G**) GB tumour samples from the FDU cohort, as well as (**H**) control brain tissue and (**I**) GB tumour samples from the HCB cohort. The classification hierarchy progresses from the innermost to the outermost radial layers: Phylum, Class, and Order. Colours correspond to viral phyla, and percentage values indicate the relative abundance within each taxonomic level. There were 159 GB tumour tissues, 21 tumour-adjacent brain tissues, and 12 control brain tissues in FDU cohort while HCB has 44 GB tumour tissues and 37 control brain tissues. 4 tumour and 11 control brain samples were excluded due to poor spectral signals.

In the FDU cohort, we identified 606 viral peptides, and 310 viral proteins derived from 71 distinct viral species (Fig. 2A-B, Supplementary File 2). Most viral peptides (414; 68.3%) were exclusively detected in tumour samples, whereas only 21 peptides (3.5%) were unique to control tissues and 42 peptides (6.9%) were exclusive to adjacent tissues (Fig. 2A). Shared peptides included 3 (0.5%) between control and adjacent tissues, 74 (12.2%) between tumour and adjacent tissues, and 16 (2.6%) between tumour and control tissues, with 36 peptides (5.9%) detected in all three tissue types.

A similar distribution pattern was observed for viral proteins. Most proteins (149; 48.1%) were uniquely detected in tumour samples, while only 3 proteins (1%) were exclusive to adjacent tissues, and none were uniquely present in control samples (Fig. 2B). Notably, 4 proteins (1.3%) were shared between control and adjacent tissues, 85 proteins (27.4%) were shared between tumour and adjacent tissues, 25 proteins (8.1%) were shared between tumour and control tissues, and 44 proteins (14.2%) were detected across all three tissue types (Fig. 2B). The higher proportion of proteins classified as unique or shared, compared with the peptide-level data, reflects the stricter criteria required for confident protein identification. Individual peptides detected in only a small number of spectra or mapping to multiple possible proteins often do not meet the thresholds for unambiguous protein assignment. Together, these findings suggest a higher frequency of viral protein detection in tumour and adjacent tissues compared to control brain samples.

In the HCB cohort, 43 peptides and 35 proteins associated with virus from 12 viral species were identified (Fig. 2C-D, Supplementary File 3). 16 peptides (37.2%) were exclusive to tumour samples, 11 peptides (25.6%) were unique to control samples, and 16 peptides (37.2%) were shared between both conditions (Fig. 2C). Similarly, for viral proteins, tumour samples exhibited a higher number of unique proteins (11, 31.4%) compared to control samples (4, 11.4%). Notably, 20 proteins were common between the tumour and control brain tissues (Fig. 2D), suggesting a subset of viral proteins consistently present across sample types.

### Distinct viral patterns across brain tissue types indicate geographical taxonomic differences in virome composition between cohorts

Next, we examined the virome composition of each cohort to determine whether cohort-specific viral profiles were present and whether these patterns might reflect geographical differences in sample origin.

In the FDU cohort, control brain tissues were predominantly composed of negative-sense RNA viruses (Negarnaviricota; 47%), primarily belonging to the Bunyavirales, Mononegavirales, and Articulavirales orders. Double-stranded DNA viruses (Peploviricota) formed the next major group, contributing 34% of the detected virome (Fig. 2E). Adjacent tissues exhibited a similar composition, with Peploviricota (45%) and Negarnaviricota (38%) as the dominant phyla (Fig. 2F). GB tumour samples had similar proportion of Peploviricota (46%) and Negarnaviricota (34%) (Fig. 2G) as the adjacent tissues. In the HCB cohort, control brain tissues were primarily composed of Peploviricota (79%), predominantly represented by Herpesvirales, while Preplasmiviricota accounted for a minor fraction (4%) (Fig. 2H). In contrast, GB tumour samples displayed fewer viral taxa, alongside a higher abundance of Peploviricota (83%) and a notable enrichment of Preplasmiviricota (13%) (Fig. 2I). In summary, comparison of viral diversity between the two cohorts revealed distinct viral phyla compositions, suggesting cohort-specific virome profiles potentially influenced by geography.

### Prevalence and distribution of viral proteins in control, adjacent, and GB tumour brain samples

**Fig. 3:**
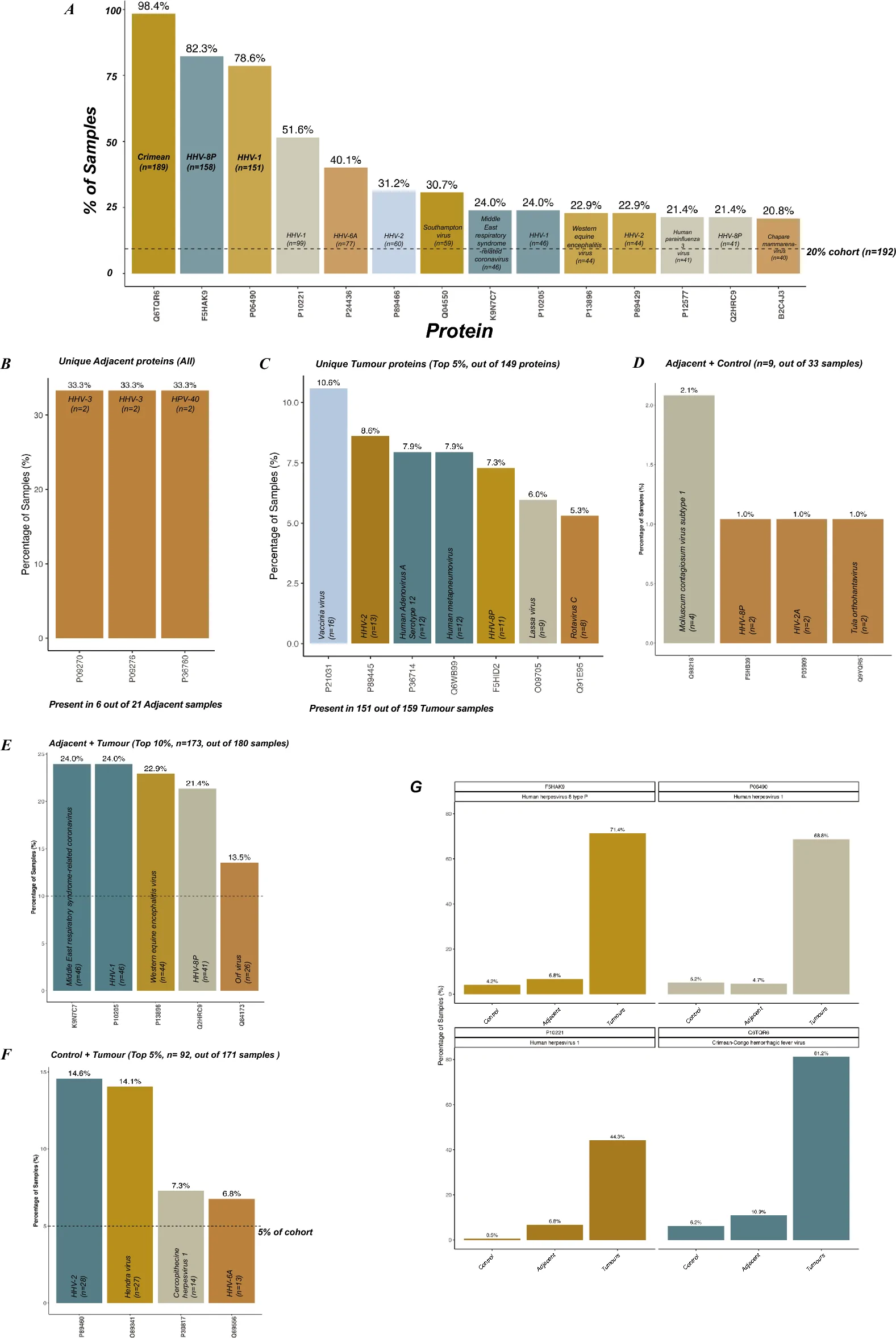
Viral protein distribution in control, adjacent and tumour brain samples from the FDU cohort. (**A**) The most prevalent viral proteins identified in more than 20% of the FDU cohort. The dashed line marks the 20% detection threshold. (**B-C**) Bar plots showing the most frequently detected unique viral proteins in (**B**) adjacent and (**C**) tumour samples. (**D-F**) Bar plots showing the most frequently detected shared viral proteins between paired tissue groups: (**D**) adjacent and control, (**E**) top 10% of viral proteins shared between adjacent and tumour tissues, and (**F**) top 5% shared between control and tumour tissues. The y-axis indicates the percentage of samples in which each protein was detected. Viral protein names and corresponding sample counts are displayed within each bar. (**G**) Stratified bar plots showing the prevalence of viral proteins detected in >50% of the total cohort, across control, adjacent, and tumour tissues. There were 159 GB tumour tissues, 21 tumour-adjacent brain tissues, and 12 control brain tissues in FDU cohort.

To further characterise the distribution of viral-associated proteins across tissue types, we assessed their prevalence in control, adjacent, and GB tumour samples within the FDU cohort. From the 310 identified viral proteins from 71 unique viral species in the public cohort, 14 proteins were detected in more than 20% of the samples belonging to 10 viral species (Fig. 3A, Supplementary File 2). Among those 14 viral proteins, 4 proteins were detected in more 50% of the entire cohort. These proteins are from Q6TAR6 (189 samples, 98.4%, Crimean-Congo hemorrhagic fever virus; CCHFV), followed by F5HAK9 (158 samples, 82.3%, HHV-8), P06490 (151 samples, 78.6%, HHV-1) and P10221 (99 samples, 51.6%, HHV-1) (Fig. 3A).

Next, we investigated the identified viral-associated proteins that are exclusive to each group. The three proteins, P09270, P09276 (HHV-3) and P36760 (HPV-40), were detected in a total of six adjacent samples (Fig. 3B). Of the 149 proteins that were exclusive to tumour, the most frequently detected proteins were P21031 (10.6%, Vaccinia virus), P89445 (8.6%, HHV-2) and P36714 (7.9%, Human Adenovirus (HAdV) A12) (Fig. 3C).

Among the viral-associated proteins shared between control and adjacent tissues, Molluscum contagiosum virus subtype 1 was detected in four samples (two control and two adjacent), while HHV-8, HIV-2, and Tula virus were each identified in two samples (one control and one adjacent per virus) (Fig. 3D). Among the shared proteins between adjacent and tumour tissues, HHV-1 and Middle East Respiratory Syndrome Coronavirus were detected in the highest number of samples, each present in 46 samples (24%) (Fig. 3E). HHV-2 (28 samples, 14.6%) and Hendra virus (27 samples, 14.1%) were found to be among the shared proteins between control and tumour (Fig. 3F).

Lastly, of the four viral proteins detected in at least 50% of the cohort (Fig. 3A), comparative analysis across control, adjacent, and tumour tissues revealed a higher prevalence of F5HAK9 (HHV-8), P06490 (HHV-1), P10221 (HHV-1), and Q6TAR6 (CCHFV) in tumour tissues (71.4, 68.8, 44.3, and 81.2% respectively) relative to adjacent and control tissues (Fig. 3G).

### Higher prevalence of HHV-1 and 2 viral proteins in tumour tissues compared to control tissues

**Fig. 4:**
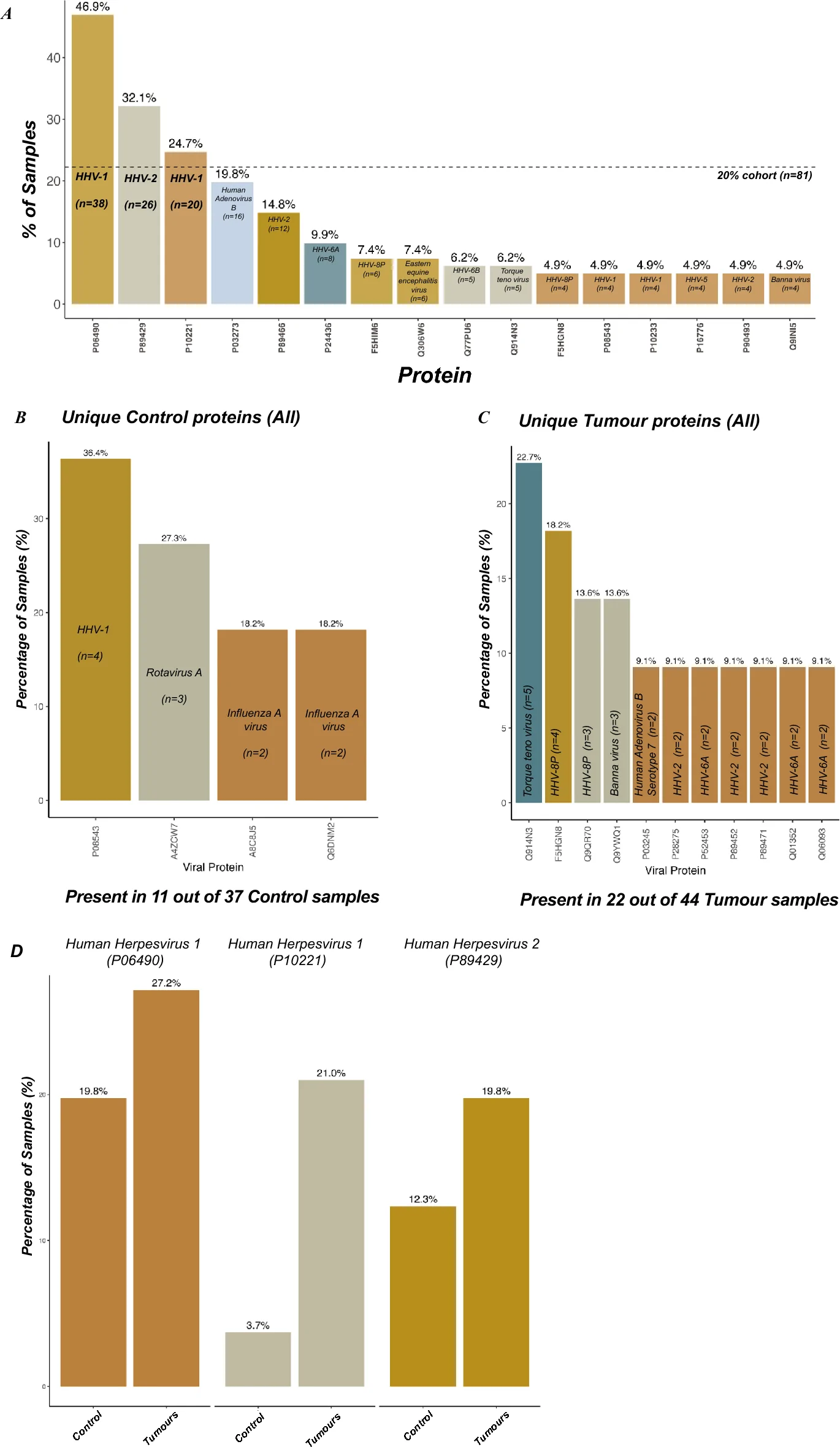
Viral protein distribution in control and tumour brain samples from the HCB cohort. (**A**) The top 16 most prevalent viral proteins identified in the HCB cohort. The dashed line marks the 20% detection threshold. (**B-C**) Bar plots showing the most frequently detected unique viral proteins in (**B**) control and (**C**) tumour samples. The y-axis indicates the percentage of samples in which each protein was detected. Viral protein names and corresponding sample counts are displayed within each bar. (**D**) Stratified bar plots showing the prevalence of viral proteins detected in more than 20% of the total cohort, across control and tumour tissues. 44 GB tumour and 37 control brain tissues from the HCB was used in this analysis. 4 tumour and 11 control brain samples were excluded due to poor spectral signals.

Detection frequency is expressed both as the number of positive samples (n) and as a percentage of the total control cohort or unless specified, allowing direct comparison of viral prevalence across samples. Among the 35 viral proteins identified from 12 viral species in the HCB cohort, three proteins were detected in over 20% of samples, indicating a subset of highly prevalent viral proteins (Fig. 4A, Supplementary File 3). These included proteins P06490 (HHV-1), P89429 (HHV-2), and P10221 (HHV-1), which were present in 46.9, 32.1, and 24.7% of samples, respectively. To support the validity of these detections, full PSM-level details for the top two HHV protein identifications, including unique peptide counts, identification probabilities, precursor mass accuracy, and per-sample detection are provided in Supplementary File 10. Annotated MS2 spectra for representative peptides from the two most prevalent proteins are provided in Supplementary File 11: MGAGAGGPK (TRM1; P06490) for HHV-1 and EILLGQMGYTEGQGVYNVVR (Portal protein UL6; P89429) for HHV-2, both co-detected within a single tumour sample (GB_002), with MGAGAGGPK identified across 5 independent samples and EILLGQMGYTEGQGVYNVVR across 4. Additional viral proteins, including those from HAdV B (19.8%), HHV-2 (14.8%), and HHV-6A (9.9%), were also observed at lower but notable frequencies.

Next, we further stratified the viral proteins that were uniquely present across tissue types. We found that 11 out of 37 control brain samples contained viral proteins that were unique to control tissues (Fig. 4B). Among these, HHV-1 had the most frequently detected protein, appearing in 36.4% of the 11 samples, followed by Rotavirus A (27.3%) and two proteins of Influenza A virus (18.2% each). Similarly, 22 out of 44 tumour samples harboured viral proteins that were uniquely found in tumour tissues (Fig. 4C). The most frequently detected tumour-specific viral proteins included Torque teno virus (22.7%), two proteins from HHV-8 (18.2 and 13.6%), and Banna virus (13.6%). These findings highlight distinct viral protein distributions in control and tumour tissues, with a greater diversity of viral proteins (notably multiple HHV proteins) identified in tumour samples.

Viral proteins detected in more than 20% of the cohort were then analysed to compare viral prevalence between control and tumour tissues. This revealed a higher presence of HHV-1 proteins (P06490 and P10221) in tumours (27.2 and 21.0%, respectively) compared to control tissues (19.8 and 3.7%, respectively). Similarly, P89429 (HHV-2) exhibited a higher frequency in tumours (19.8%) than in control tissues (12.3%) (Fig. 4D).

### Differentially expressed host proteins between GB tumours and control brain tissues

As viruses can promote cellular transformation by modulating host pathways, analysing the human proteome provides complementary insight into how viral protein presence may influence tumour biology. To explore this, we compared the proteomic profiles of 48 tumour specimens and 48 control specimens using a DIA approach enhanced by a recombinant protein DDA library^21^. The differences in the number of samples between meta-proteomic (viral peptides) and proteome (human peptides) analysis is due to the abundance of peptides. Viral peptides are present at much lower levels than human peptides. As a result, some samples with adequate human proteome signal do not meet the minimum detection threshold for viral analysis, whereas all samples pass quality control for human proteomics.

**Fig. 5:**
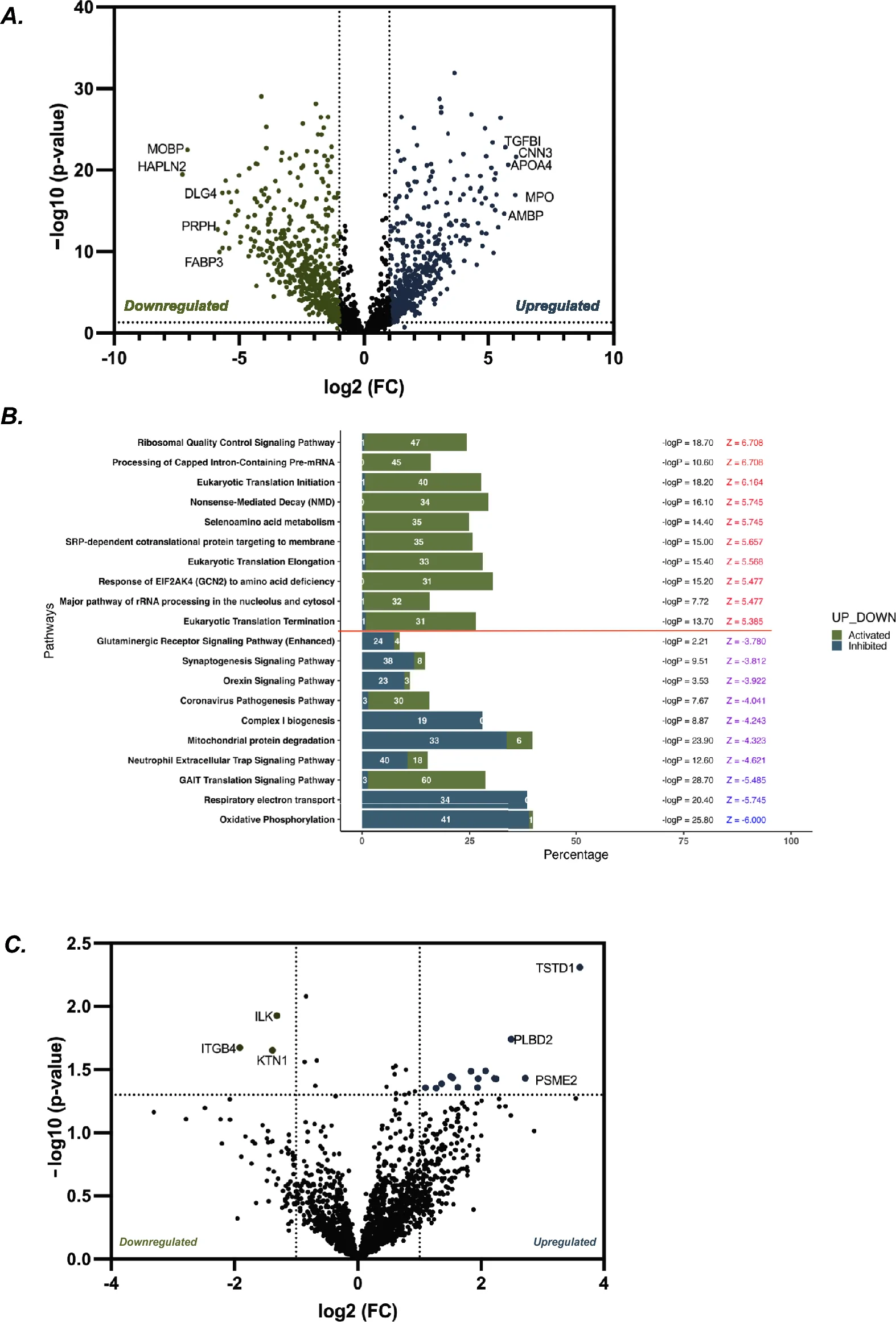
Differential proteomic profiling and pathway analysis in GB tumours from HCB cohort. (**A**) Volcano plots of up and down-regulated human proteins in GB tumour samples, as compared to control. Dotted lines on the X- axis are log2 FC = −1 and 1. (**B**) This bar plot presents the 10 most upregulated (positive Z-score) and 10 most downregulated (negative Z-score) pathways identified through Ingenuity pathway analysis (IPA). Pathways were selected based on statistical significance (p < 0.05) and a Z-score threshold of ≥ +2, indicating a strong predicted activation or downregulation. The x-axis represents the percentage of genes involved in each pathway, while the y-axis lists the pathways. Activated pathways (Z > 2) are shown in blue and are associated with increased pathway activity. Inhibited pathways (Z < -2) are shown in green and represent suppressed or inhibited activity. Each bar is annotated with the number of genes contributing to the pathway. 48 GB tumour and 48 control brain tissues were included in the human proteome analysis. (**C**) Differential proteomic profiling in GB stratified by HHV-1 status from HCB cohort. Volcano plots of up and down-regulated human proteins in GB tumour samples, as compared to control. Dotted lines on the X- axis are log2 FC = −1 and 1. 11 HHV-1 positive and 11 HHV-1 negative tissues used in this comparison.

A total of 1874 proteins were quantified (Supplementary File 4), of which 1023 were differentially expressed proteins (DEPs) between control and GB samples. Among the 1023 DEPs, 511 proteins were upregulated and 512 were downregulated in tumour samples (Fig. 5A). Calponin-3 (CNN3) was the most significantly upregulated protein (FC of 6.11), followed by Myeloperoxidase (MPO), Apolipoprotein A4 (APOA4), Transforming Growth Factor Beta Induced (TGFBI), and Alpha-1-Microglobulin/Bikunin Precursor (AMBP). Hyaluronan and proteoglycan link protein 2 (HAPLN2) was the most downregulated protein (FC of 7.27) followed by Myelin Associated Oligodendrocyte Basic Protein (MOBP), Peripherin (PRPH), Fatty Acid Binding Protein 3 (FABP3), and Discs Large MAGUK Scaffold Protein 4 (DLG4). These 10 DEPs were also statistically validated using the Mann-Whitney U test, further reinforcing the robustness of these findings (Supplementary File 5). To assess robustness, analyses were repeated after exclusion of a small number of samples identified as potential technical outliers during pre-normalisation quality control. The reanalysis fully recapitulated all original DEPs while identifying additional proteins, consistent with increased analytical sensitivity, without altering the primary conclusions (Supplementary File 6).

To understand the biological relevance of the observed changes in the tissue proteome, we next performed further pathway analysis using Ingenuity Pathway Analysis (IPA). The analysis revealed 216 differentially regulated pathways between control and GB (Supplementary File 7), with 119 pathways were activated and 97 pathways were found to be inhibited (p-value ≤ 0.05, Z-score ±2) in GB (Fig. 5B). Activated pathways were predominantly associated with RNA processing, mRNA stability, and protein translation, consistent with the elevated translational activity reported in high-grade gliomas^33–35^. Signatures of cell-cycle progression, DNA replication, and telomerase activity were also enriched, reflecting the proliferative phenotype characteristic of GB^36^. Several innate immune and NF-κB-related processes showed activation (Supplementary File 7), in line with prior studies demonstrating inflammatory pathway engagement and microenvironmental remodelling in GB^37,38^.

In contrast, inhibited pathways were strongly enriched for mitochondrial respiration, TCA cycle activity, and oxidative phosphorylation, recapitulating the metabolic reprogramming and reduced respiratory capacity typical of GB^39–41^. Downregulation of synaptic, neurotransmission, and neuronal differentiation pathways was also observed, consistent with the loss of neuronal signatures that accompanies tumour infiltration and cellular state transitions in GB^42,43^. Together, these coordinated pathway-level changes reflect enhanced biosynthetic and proliferative programs alongside suppression of mitochondrial and neuronal functions in GB.

## Correlations between viral proteins and human protein expressions

Having established distinct viral and human proteomics profiles between GB and non-cancerous tissues, we next examined whether viral protein abundance was with corresponding changes in host protein expression in the HCB cohort. To characterise the relationship between viral protein presence and host protein expression, we performed correlation analyses across tissue groups. However, due to the limited sample size within each comparison group, correlation analyses could not be performed on either exclusive or shared viral proteins in the cohort. Therefore, a point-biserial correlation analysis was performed to examine associations between the presence of viral proteins and host protein expression.

Top three viruses (HHV-1, HHV-2, and HAdV-7) that appeared in more than 20% of the HCB cohort was selected for exploratory correlation analysis (44 GB tumour and 37 control brain tissues, Supplementary Files 3 and 8), based on peptide-level detection across samples. This yielded 12 viral proteins, for which a binary presence--absence matrix (1 = present, 0 = absent) was generated. HHV-1 proteins were detected in 32 tumour and 23 control brain tissues, HHV-2 proteins in 24 tumour and 14 control tissues, and HAdV-7 proteins in 15 tumour and 3 control tissues (Supplementary File 3). This matrix was then integrated with raw expression data for the quantified 1874 human proteins. This revealed 314 significant associations (FDR-adjusted p < 0.05), pointing to potential interactions between virus and host proteins (Supplementary File 8). Following multiple testing correction, we highlight the top five positively and negatively correlated viral-host protein pairs. Given the relatively small number of virus-positive samples for the selected viral proteins, these analyses should be considered exploratory and were intended to identify potential virus-host associations rather than establish robust mechanistic relationships.

The top positive correlations were observed between viral proteins and human proteins. The HAdV-7 early protein, E1B 55 kDa exhibited the strongest correlations with host protein Desmin (DES; r = 0.96), Lactate Dehydrogenase A (LDHA; r = 0.74), and Ribosomal protein L15 (RPL15; r = 0.71), suggesting potential involvement in cytoskeletal modulation, metabolic rewiring, and translation, respectively. Additionally, HHV-2 DNA primase was positively associated with both Zinc Finger Protein 268 (ZNF268; r = 0.78), and Hemoglobin subunit beta (HBB; r = 0.72) (Table 2).

**Table 2:** Top 5 positively (highlighted in blue) and negative (highlighted in green) correlations between viral proteins and human proteins. HAdV-7 = Human adenovirus type 7; HHV-2 = Human herpesvirus 2; DES = Desmin; ZNF268 = Zinc finger protein 268; LDHA = lactate dehydrogenase A; HBB = Hemoglobin subunit beta; RPL15 = Ribosomal protein L15; NDUFB3 = NADH dehydrogenase 1 beta subcomplex subunit 3; CKB = Creatine kinase B-type; MAG = Myelin-associated glycoprotein; ANXA11 = Annexin A11; ANXA6 = Annexin A6.

Uniprot viral protein	Virus	Viral protein	Human protein	Uniprot human protein	Correlation	Adjusted-P
P03245	HAdV-7	E1B 55 kDa protein (large T-antigen)	DES	P17661	0.95933936	p<0.0001
P89471	HHV-2	DNA primase	ZNF268	Q14587	0.7810834	p<0.0001
P03245	HAdV-7	E1B 55 kDa protein (large T-antigen)	LDHA	P00338	0.73852853	p<0.0001
P89471	HHV-2	DNA primase	HBB	P68871	0.72305048	p<0.0001
P03245	HAdV-7	E1B 55 kDa protein (large T-antigen)	RPL15	P61313	0.70601802	p<0.0001
P03273	HAdV-7	Packaging protein 1	NDUFB3	O43676	−0.4071852	p=0.014
P10221	HHV-2	Inner tegument protein	CKB	P12277	−0.4082917	p=0.014
P10221	HHV-2	Inner tegument protein	MAG	P20916	−0.4120894	p=0.012
P03273	HadV-7	Packaging protein 1	ANXA11	P50995	−0.4170677	p=0.01
P06490	HHV-2	Tripartite terminase subunit 1	ANXA6	P08133	−0.4480511	p=0.003

In contrast, the top negative correlations were observed between viral structural proteins and host proteins involved in mitochondrial function, energy metabolism, and neuronal integrity. The top five negative correlations included HAdV Packaging protein 1 with NADH dehydrogenase 1 beta subcomplex subunit 3 (NDUFB3; r = -0.41) and Annexin A11 (ANXA11; r = -0.42). HHV-2 Inner Tegument Protein were negatively correlated with creatine kinase B-type (CKB; r = -0.41) and Myelin-Associated Glycoprotein (MAG; r = -0.41) and Annexin A6 (ANXA6; r = -0.45) (Table 2).

## Differential protein expression in HHV-1-positive versus HHV-1-negative tumours

Building on our integrated metaproteomic dataset, we next examined whether the detection of a specific virus within tumour tissues corresponded with measurable differences in host protein expression. Among the viral species identified, HHV-1 was most frequently detected in GB samples from HCB cohort. Due to sample size limitations, this exploratory analysis was limited to a small subset of HHV-1 positive and negative tumour samples and should therefore be interpreted cautiously. To ensure an unbiased comparison, the HHV-1 positive group was down sampled to match the number of available HHV-1 negative tumours (n = 11 per group). Differential expression analysis was conducted on tumour tissues stratified by HHV-1 status based on our metaproteome analysis. A total of 2060 proteins were quantified, of which 18 were significantly dysregulated (adjusted p ≤ 0.05; fold change ± 2). Of these, 15 proteins were upregulated in HHV-1 positive tumours, including Thiosulfate Sulfurtransferase Like Domain Containing 1 (TSTD1; FC of 3.61), Proteasome Activator Subunit 2 (PSME2; FC of 2.73) and Phospholipase B Domain Containing 2 (PLBD2; FC of 2.50). In contrast, Integrin Subunit Beta 4 (ITGB4; FC of 1.92), Kinectin 1 (KTN1; FC of 1.38) and Integrin Linked Kinase (ILK; FC of 1.31) were downregulated in HHV-1 positive tumours, indicating enrichment in HHV-1 negative tumours. Together, these findings suggest distinct proteomic alterations between HHV-1-positive and HHV-1-negative tumours, with differential abundance in proteins related to mitochondrial activity, protein turnover, and cell adhesion/signalling (Figure 5C Supplementary File 9).

## Discussion

This study builds on existing molecular and genomic evidence of viral presence in GB by applying a metaproteomic approach in identifying viral proteins and their associations with host proteomic alterations. The FDU cohort showed a higher number of detected viral species (71 viral species) than the HCB cohort (12 viral species), a difference likely influenced by the cohort size, sampling depth and technical factors related to sample processing and detection. Notably, all 12 viral species identified in the HCB cohort were also present in the FDU cohort, suggesting shared viral presence across datasets despite differences in scale and methodology. In addition, geographical viral variation^44^ may further contribute to differences in viral composition across tumour samples. As demonstrated in HPV-related cancers, the attributable fraction of infection-driven cancers differs significantly by region- ranging from under 3% in Australia and the USA to over 20% in India and sub-Saharan Africa^45^.

RNA-directed RNA polymerase L from CCHFV was the most prevalent viral protein identified in the FDU cohort but was notably absent from our HCB cohort. Additional unexpected viral proteins, including those associated with Hendra virus and Middle East Respiratory Syndrome Coronavirus, were also detected in the FDU cohort. Given the unexpected nature of these findings in brain tissues, these observations should be interpreted cautiously, as they may reflect low-abundance false-positive peptide matching and/or environmental or procedural contamination. CCHFV is a tick-borne virus endemic to certain regions of China, which corresponds to the geographical origin of the FDU cohort^46,47^. In contrast to this cohort-specific viral finding, both datasets showed evidence of HHV-1 and HHV-2, albeit at different frequencies. Our HCB samples, collected in Australia, revealed a high prevalence of proteins from HHV-1 and HHV-2. This pattern is consistent with seroepidemiological data showing that 70-80% of Australians have been exposed to HHV-1 and approximately 12% for HHV-2^48,49^. Similarly, seroprevalence studies in China have reported a very high rate of HHV-1 infection (92.0%) and a lower rate of HHV-2 infection (13.2%)^50^, which aligns with observed proportions in the FDU dataset. Together, these observations suggest that geographical variation in viral circulation influences the virome landscape across cohorts while ubiquitous HHV such as HHV-1 and HHV-2 appear consistently across populations. Further studies are needed to confirm these geographic associations. We also acknowledge that the differences in protein extraction methods and lab-specific workflows can introduce variability in protein and viral identifications^51^.

It is noteworthy that HHVs constituted the majority of viral proteins detected in both cohorts. This may reflect the widespread prevalence, neurotropic nature, and latent persistence of HHVs within neural tissues, rather than tumour-specific viral enrichment. While HHV proteins were more frequently detected in tumour tissues in our cohorts, these observations should be interpreted cautiously as differences in viral detection frequency and do not establish active infection or causative involvement in GB. HHV-1 was the most abundant viral species in both cohorts, a finding supported by the detection of two HHV-1 proteins (P06490, tripartite terminase subunit 1) and P10221 (inner tegument protein), with HHV-2 being the next most frequently observed. These proteins function in virion maturation and assembly during the lytic phase^52,53^, but their detection alone does not confirm active viral replication or reactivation. There is also no evidence that these HHV-1 proteins contribute to oncogenic transformation, and further investigation is required to determine their biological relevance. Additional HHVs, including HHV-6A and HHV-8, were also detected across both datasets. While individual herpesviruses have previously been reported in brain tissues, concurrent presence of HHV-1 and HHV-2 within GB tumour samples has not been widely documented. The detection of multiple herpesviruses is consistent with the their high prevalence of in the human population and their known neurotropic and latent nature^54^. While their presence within GB tissues does not demonstrate a causal role in tumour progression, it is consistent with reports that the tumour microenvironment can be permissive for the persistence of latent or opportunistic viruses through local immune modulation^55,56^. Importantly, these observations should not be interpreted as evidence of causality. Additional functional studies are required to determine whether HHVs play any role in GB.

Proteomic pathway analysis revealed extensive host remodelling characterised in the GB tumour tissues compared to control brain tissues by metabolic reprogramming, neurodegeneration-related processes, and immune dysregulation, consistent with previous studies^33–36,57^. We observed alterations in immune-related pathways, including enrichment of pattern-recognition receptor and type I interferon signalling, together with suppression of antigen presentation and nucleic acid-sensing pathways such as MHC class II processing and cGAS-STING signalling. Specifically, enrichment of pattern-recognition receptor and type I interferon signalling is consistent with an active host response to viral material, as described for HHV and cytomegalovirus in GB^13,14,58^. In contrast, suppression of antigen presentation and nucleic acid-sensing pathways, including MHC class II processing and cGAS-STING signalling, may contribute to an immunosuppressed microenvironment that supports tumour survival and is permissive to viral persistence^59,60^. While these interpretations are based on pathway-level inference, they support a model of viral accommodation rather than direct oncogenic causality, although further studies are required to establish mechanistic relevance.

In addition to the pathway alterations identified by IPA, the correlation analysis revealed several associations between viral and human proteins (Table 2), suggesting a potential contribution of viral proteins to tumour-associated biological processes. The E1B 55 kDa protein showed positive correlations with DES, LDHA, and RPL15. Adenoviral E1B 55 kDa protein is a multifunctional phosphoprotein that regulates adenoviral replication^61^ and mediates the degradation of several host proteins, including p53^62,63^. The association with DES is consistent with prior evidence that adenoviruses, including E1B-containing complexes, modulate host cytoskeletal architecture and intracellular trafficking to facilitate viral replication and assembly^64,65^. The correlation with LDHA, a key glycolytic enzyme^66,67^, aligns with prior observations that viral infection enhances glycolysis to fulfil the biosynthetic demands of viral replication or cellular transformation^68–70^. Similarly, the association with RPL15, a component of the 60S ribosomal subunit, is consistent with evidence that adenoviruses exploit ribosome-associated factors to prioritise translation of viral mRNA^71^. Extending these observations, HHV-2 DNA primase UL52 also showed strong positive associations with ZNF268, a KRAB-domain transcriptional repressor involved in NF-κB signalling and immune regulation^72–74^. Herpesviruses are known to subvert host transcriptional and epigenetic regulatory mechanisms to suppress antiviral immune signalling and create a cellular environment that is permissive for efficient viral replication^75,76^. The observed association between UL52 and HBB, although unexpected, may arise from stress-associated microenvironmental or blood-derived contributions within the highly vascularised and oxidative GB tumour milieu. Such an association is consistent with reports of ectopic haemoglobin induction under oxidative stress in non-erythroid tissues outside the brain^77,78^. However, further investigation will be required to substantiate this relationship.

Across multiple viral proteins, inverse associations were observed with host factors involved in cellular metabolism, membrane trafficking, and neural structural integrity. For HAdV-7 packing protein 1, negative associations with the mitochondrial complex I subunit NDUFB3 and the annexin ANXA11 are consistent with viral strategies that suppress oxidative phosphorylation^79^ in favour of glycolytic reprogramming^80–85^, thereby supporting the bioenergetic demands of viral assembly^68,86,87^, as well as modulation of calcium-dependent membrane trafficking and stress-response pathways commonly targeted during viral replication and assembly^88–90^. Similarly, associations involving HHV-2 tegument and packaging proteins implicated host pathways related to energy homeostasis and neurostructural maintenance, including reduced association with CKB, consistent with herpesvirus-driven metabolic reprogramming that redirects host energy resources to support viral replication^91,92^, and with MAG, suggesting perturbation of axon-glial signalling and myelin maintenance^93^. In addition, the negative association between the HHV-2 tripartite terminase subunit 1 and ANXA6, a regulator of endosomal trafficking and exosome release^94^ with reported antiviral and tumour-suppressive roles activity^94–97^, may reflect viral interference with host defence or vesicular transport pathways. Collectively, these patterns align with established DNA virus strategies that reshape host metabolic, membrane, and structural processes to facilitate viral persistence rather than direct oncogenic transformation. However, these associations do not provide direct mechanistic evidence, and further studies are needed to examine this relationship.

There are several limitations to our study. In the absence of viral contamination testing in the operating theatre and pathology laboratory where tissues were collected and processed, we cannot conclusively determine that the viral proteins detected originated exclusively from the tissues itself. This is particularly important given the low-abundance nature of viral peptides and the retrospective use of archival FFPE samples, across which contamination may arise at multiple stages including surgical handling, tissue retrieval, pathology processing, FFPE fixation and storage, biobank handling, and mass spectrometry workflows, none of which could be systematically controlled or reassessed. This study was also designed as an exploratory discovery-based analysis rather than a targeted viral proteomics workflow as the expected viral species and abundance were not known beforehand, it was not feasible to generate virus-specific spectral libraries or perform spike-in benchmarking using synthetic viral peptides, and no validated virus-negative brain tissue construct was available as a true negative control. Additionally, large metaproteomic database searches increase the probability of spurious PSM, and sequence homologues in organisms absent from the search database cannot be excluded; identifications resting on a single unique peptide are particularly susceptible to these artefacts and should be regarded as hypothesis-generating rather than confirmatory. Mass spectrometry-based detection is further subject to sensitivity and dynamic range constraints, with extremely low-abundance signals potentially remaining below detection thresholds. The lack of matched normal tissues and the limited number of samples with unique viral proteins also constrained our ability to perform robust correlation analyses between viral and host protein expression.

All viral detections should therefore be interpreted as evidence of viral-associated peptide detection rather than definitive evidence of active infection or biological viral burden. Orthogonal validation, including immunohistochemistry, targeted mass spectrometry, or in situ hybridisation will be required to confirm cellular localisation, specificity, and biological relevance within the GB microenvironment. Future investigations should incorporate genomic and transcriptomic profiling to assess whether specific tumour or immune cell populations preferentially harbour viral proteins.

In conclusion, this study demonstrated that, despite geographical differences between cohorts, HHVs remain the predominant viral species detected across independent GB cohorts. Although our study did not establish direct functional interactions between host and viral proteins, the observed correlations between the viral and host proteins, alongside pathways alterations identified by IPA, suggest a potential association between HHVs and GB. This interpretation is supported by previous reports demonstrating functional interactions between HHVs and human signalling pathways^98–103^. It is important to acknowledge that the presence of these viruses may be opportunistic rather than causative, in line with the “hit and run” hypothesis, whereby tumour development creates an immunosuppressive microenvironment that permits viral persistence or reactivation within the local milieu. Nonetheless, these findings reinforce the potential relevance of viral presence in GB pathology and warrant further investigation. If future studies establish a functional role for viral activity in GB progression, this may open avenues to explore antiviral strategies as an adjunct to existing therapeutic approaches.

## Supplementary Information

Supplementary Information 1.

Supplementary Information 2.

Supplementary Information 3.

Supplementary Information 4.

Supplementary Information 5.

Supplementary Information 6.

Supplementary Information 7.

Supplementary Information 8.

Supplementary Information 9.

Supplementary Information 10.

Supplementary Information 11.

## Data Availability

Publicly available datasets from the FDU cohort can be found in ProteomeXchange Consortium with the identifier PXD038732 20 . The metaproteomic and proteomic mass spectrometry data have been deposited to the ProteomeXchange Consortium via the PRIDE 103 partner repository with the following project accession numbers: PXD074714 (DDA) and PXD075144 (DIA). All other relevant data supporting the findings of this study are included in the main article and Supplementary Information. Additional information is available from the corresponding author upon reasonable request.

## Abbreviations

AMBP: Alpha-1-microglobulin/bikunin precursor
ANXA6: Annexin A6
APOA4: Apolipoprotein A4
ATP5ME: ATP synthase membrane subunit E
AUC: Area under the curve
BORCS7: BLOC-1 related complex subunit 7
BSCL2: BSCL2 lipid droplet biogenesis associated, seipin
CCHFV: Crimean-congo hemorrhagic fever virus
CNN3: Calponin-3
DDA: Data-dependent acquisition
DDX1: DEAD-box helicase 1
DDX17: DEAD-box helicase 17
DEPs: Differentially expressed proteins
DES: Desmin
DIA: Data independent acquisition
DLG4: Discs large MAGUK scaffold protein 4
EGFR: Epidermal growth factor receptor
ELISA: Enzyme-linked immunosorbent assay
FABP3: Fatty acid binding protein 3
FDU: Fudan university
FFPE: Formalin-fixed, paraffin-embedded
FLNC: Filamin C
GB: Glioblastoma
GLS: Glutaminase
GPX4: Glutathione peroxidase 4
HAdV: Human adenovirus
HAPLN2: Hyaluronan and proteoglycan link protein 2
HBB: Hemoglobin subunit beta
HCB: Hunter cancer biobank
HHV: Human herpesvirus
HPV: Human papillomavirus
IDH: Isocitrate dehydrogenase
IHC: Immunohistochemistry
ILK: Integrin linked kinase
IPA: Ingenuity pathway analysis
ITGB4: Integrin subunit beta 4
KTN1: Kinectin 1
LDHA: L-lactate dehydrogenase A chain
MAG: Myelin-associated glycoprotein
MOBP: Myelin associated oligodendrocyte basic protein
MPO: Myeloperoxidase
MS: Mass spectrometry
MYH9: Myosin heavy chain 9
NDUFB3: NADH dehydrogenase 1 beta subcomplex subunit 3
NGS: Next-generation sequencing
PHF24: PHD finger protein 24
PLBD2: Phospholipase B domain containing 2
PRPH: Peripherin
PSM: Peptide-spectrum match
PSME2: Proteasome activator subunit 2
RABIF: RAB interacting factor
REEP1: Receptor accessory protein 1
ROC: Receiver operating characteristic
ROCK2: Rho associated coiled-coil containing protein kinase 2
RPL15: Large ribosomal subunit protein L15
SLC6A9: Solute carrier family 6 member 9
SYN3: Synapsin III
TERT: Telomerase reverse transcriptase
TGFBI: Transforming growth factor beta induced
TPP: Trans-proteomic pipeline
TSTD1: Thiosulfate sulfurtransferase like domain containing 1
ZNF268: Zinc finger protein 268

## References

[1] Louis, D. N. et al. The 2021 WHO Classification of tumors of the central nervous system: a summary. *Neuro Oncol.* **23**(8), 1231–51 (2021). 34185076 10.1093/neuonc/noab106 PMC8328013

[2] Ostrom, Q. T. et al. CBTRUS statistical report: primary brain and central nervous system tumors diagnosed in the United States in 2006–2010. *Neuro Oncol.* **15**, ii1-56 (2013). 24137015 10.1093/neuonc/not151 PMC3798196

[3] Louis, D. N. et al. cIMPACT-NOW update 6: new entity and diagnostic principle recommendations of the cIMPACT-Utrecht meeting on future CNS tumor classification and grading. *Brain Pathol.* **30**(4), 844–56 (2020). 32307792 10.1111/bpa.12832 PMC8018152

[4] Brat, D. J. et al. cIMPACT-NOW update 3: recommended diagnostic criteria for “Diffuse astrocytic glioma, IDH-wildtype, with molecular features of glioblastoma, WHO grade IV”. *Acta Neuropathol.* **136**(5), 805–10 (2018). 30259105 10.1007/s00401-018-1913-0 PMC6204285

[5] Akhtar, S., Vranic, S., Cyprian, F. S. & Al Moustafa, A. E. Epstein-barr virus in gliomas: cause, association, or artifact?. *Front Oncol.* **8**, 123 (2018). 29732319 10.3389/fonc.2018.00123 PMC5919939

[6] Cohen, K. J. et al. Temozolomide in the treatment of high-grade gliomas in children: a report from the children’s oncology group. *Neuro Oncol.* **13**(3), 317–23 (2011). 21339192 10.1093/neuonc/noq191 PMC3064602

[7] Ostrom, Q. T. et al. The epidemiology of glioma in adults: a “state of the science” review. *Neuro Oncol.* **16**(7), 896–913 (2014). 24842956 10.1093/neuonc/nou087 PMC4057143

[8] Hanif, F., Muzaffar, K., Perveen, K., Malhi, S. M. & Simjee, Sh. U. Glioblastoma multiforme: a review of its epidemiology and pathogenesis through clinical presentation and treatment. *Asian Pac. J. Cancer Prev.* **18**(1), 3–9 (2017). 28239999 10.22034/APJCP.2017.18.1.3 PMC5563115

[9] Munger, K., Phelps, W. C., Bubb, V., Howley, P. M. & Schlegel, R. The E6 and E7 genes of the human papillomavirus type 16 together are necessary and sufficient for transformation of primary human keratinocytes. *J Virol.* **63**(10), 4417–21 (1989). 2476573 10.1128/jvi.63.10.4417-4421.1989 PMC251060

[10] Doorbar, J. et al. The biology and life-cycle of human papillomaviruses. *Vaccine.* **30**(Suppl 5), F55-70 (2012). 23199966 10.1016/j.vaccine.2012.06.083

[11] Dyson, N., Howley, P. M., Munger, K. & Harlow, E. The human papilloma virus-16 E7 oncoprotein is able to bind to the retinoblastoma gene product. *Science.* **243**(4893), 934–7 (1989). 2537532 10.1126/science.2537532

[12] Gunasegaran, B., Ashley, C. L., Marsh-Wakefield, F., Guillemin, G. J. & Heng, B. Viruses in glioblastoma: an update on evidence and clinical trials. *BJC Rep.* **2**(1), 33 (2024). 39516641 10.1038/s44276-024-00051-z PMC11524015

[13] Cobbs, C. S. et al. Human cytomegalovirus infection and expression in human malignant glioma. *Cancer Res.* **62**(12), 3347–50 (2002). 12067971

[14] Mitchell, D. A. et al. Sensitive detection of human cytomegalovirus in tumors and peripheral blood of patients diagnosed with glioblastoma. *Neuro Oncol* **10**(1), 10–8 (2008). 17951512 10.1215/15228517-2007-035 PMC2600830

[15] Aguiar-Pulido, V. et al. Metagenomics, metatranscriptomics, and metabolomics approaches for microbiome analysis. *Evol. Bioinform. Online* **12**(Suppl 1), 5–16 (2016). 27199545 10.4137/EBO.S36436 PMC4869604

[16] He, S., Chakraborty, R. & Ranganathan, S. Metaproteomic analysis of an oral squamous cell carcinoma dataset suggests diagnostic potential of the mycobiome. *Int. J. Mol. Sci.* 10.3390/ijms24021050 (2023). 36674563 PMC9865486

[17] DeGruttola, A. K., Low, D., Mizoguchi, A. & Mizoguchi, E. Current understanding of dysbiosis in disease in human and animal models. *Inflamm. Bowel Dis.* **22**(5), 1137–50 (2016). 27070911 10.1097/MIB.0000000000000750 PMC4838534

[18] Hou, K. et al. Microbiota in health and diseases. *Signal Transduct. Target. Ther.* **7**(1), 135 (2022). 35461318 10.1038/s41392-022-00974-4 PMC9034083

[19] Afzaal, M. et al. Human gut microbiota in health and disease: unveiling the relationship. *Front Microbiol.* **13**, 999001 (2022). 36225386 10.3389/fmicb.2022.999001 PMC9549250

[20] Wang, Y. et al. Proteogenomics of diffuse gliomas reveal molecular subtypes associated with specific therapeutic targets and immune-evasion mechanisms. *Nat Commun.* **14**(1), 505 (2023). 36720864 10.1038/s41467-023-36005-1 PMC9889805

[21] Ahn, S. B. et al. Use of a recombinant biomarker protein DDA library increases DIA coverage of low abundance plasma proteins. *J. Proteome Res.* **20**(5), 2374–89 (2021). 33752330 10.1021/acs.jproteome.0c00898

[22] Rappsilber, J., Mann, M. & Ishihama, Y. Protocol for micro-purification, enrichment, pre-fractionation and storage of peptides for proteomics using StageTips. *Nat. Protoc.* **2**(8), 1896–906 (2007). 17703201 10.1038/nprot.2007.261

[23] Kong, A. T., Leprevost, F. V., Avtonomov, D. M., Mellacheruvu, D. & Nesvizhskii, A. I. MSFragger: ultrafast and comprehensive peptide identification in mass spectrometry-based proteomics. *Nat. Methods.* **14**(5), 513–20 (2017). 28394336 10.1038/nmeth.4256 PMC5409104

[24] Demichev, V., Messner, C. B., Vernardis, S. I., Lilley, K. S. & Ralser, M. DIA-NN: neural networks and interference correction enable deep proteome coverage in high throughput. *Nat. Methods.* **17**(1), 41–4 (2020). 31768060 10.1038/s41592-019-0638-x PMC6949130

[25] He, S. & Ranganathan, S. Bioinformatic analysis to investigate metaproteome composition using trans-proteomic pipeline. *Curr. Protoc.* **2**(7), e506 (2022). 35862176 10.1002/cpz1.506

[26] Deutsch, E. W. et al. Trans-proteomic pipeline: robust mass spectrometry-based proteomics data analysis suite. *J. Proteome. Res.* **22**(2), 615–24 (2023). 36648445 10.1021/acs.jproteome.2c00624 PMC10166710

[27] Li, R. & Bilal, U. Interactive web-based data visualization with R, plotly, and shiny. *Biometrics.* **77**(2), 776–7 (2021).

[28] Liu, M. & Dongre, A. Proper imputation of missing values in proteomics datasets for differential expression analysis. *Brief. Bioinform.* 10.1093/bib/bbaa112 (2021). 32520347

[29] Wu, J. X. et al. SWATH mass spectrometry performance using extended peptide MS/MS assay libraries. *Mol. Cell Proteomics.* **15**(7), 2501–14 (2016). 27161445 10.1074/mcp.M115.055558 PMC4937520

[30] Pascovici, D., Handler, D. C., Wu, J. X. & Haynes, P. A. Multiple testing corrections in quantitative proteomics: a useful but blunt tool. *Proteomics* **16**(18), 2448–53 (2016). 27461997 10.1002/pmic.201600044

[31] Tyanova, S. et al. The Perseus computational platform for comprehensive analysis of (prote)omics data. *Nat. Methods.* **13**(9), 731–40 (2016). 27348712 10.1038/nmeth.3901

[32] Bjelosevic, S. et al. Quantitative age-specific variability of plasma proteins in healthy neonates children and adults. *Mol. Cell Proteomics.* **16**(5), 924–35 (2017). 28336724 10.1074/mcp.M116.066720 PMC5417830

[33] DeSouza, P. A. et al. Long, noncoding RNA dysregulation in glioblastoma. *Cancers (Basel)* 10.3390/cancers13071604 (2021). 33807183 PMC8037018

[34] Montiel-Davalos, A., Ayala, Y. & Hernandez, G. The dark side of mRNA translation and the translation machinery in glioblastoma. *Front Cell Dev. Biol.* **11**, 1086964 (2023). 36994107 10.3389/fcell.2023.1086964 PMC10042294

[35] Kim, J. H. et al. SON drives oncogenic RNA splicing in glioblastoma by regulating PTBP1/PTBP2 switching and RBFOX2 activity. *Nat. Commun.* **12**(1), 5551 (2021). 34548489 10.1038/s41467-021-25892-x PMC8455679

[36] Brennan, C. W. et al. The somatic genomic landscape of glioblastoma. *Cell* **155**(2), 462–77 (2013). 24120142 10.1016/j.cell.2013.09.034 PMC3910500

[37] Mathur, R. et al. Glioblastoma evolution and heterogeneity from a 3D whole-tumor perspective. *Cell* **187**(2), 446–63 e16 (2024). 38242087 10.1016/j.cell.2023.12.013 PMC10832360

[38] Bhat, K. P. L. et al. Mesenchymal differentiation mediated by NF-κB promotes radiation resistance in glioblastoma. *Cancer Cell* **24**(3), 331–46 (2013). 23993863 10.1016/j.ccr.2013.08.001 PMC3817560

[39] Reznik, E., Wang, Q., La, K., Schultz, N. & Sander, C. Mitochondrial respiratory gene expression is suppressed in many cancers. *Elife* 10.7554/eLife.21592 (2017). 28099114 PMC5243113

[40] Venneti, S. & Thompson, C. B. Metabolic reprogramming in brain tumors. *Annu. Rev. Pathol.* **12**, 515–45 (2017). 28068482 10.1146/annurev-pathol-012615-044329

[41] Seyfried, T. N., Flores, R. E., Poff, A. M. & D’Agostino, D. P. Cancer as a metabolic disease: implications for novel therapeutics. *Carcinogenesis* **35**(3), 515–27 (2014). 24343361 10.1093/carcin/bgt480 PMC3941741

[42] Patel, A. P. et al. Single-cell RNA-seq highlights intratumoral heterogeneity in primary glioblastoma. *Science* **344**(6190), 1396–401 (2014). 24925914 10.1126/science.1254257 PMC4123637

[43] De Silva, M. I., Stringer, B. W. & Bardy, C. Neuronal and tumourigenic boundaries of glioblastoma plasticity. *Trends Cancer* **9**(3), 223–36 (2023). 36460606 10.1016/j.trecan.2022.10.010

[44] Zuo, T. et al. Human-gut-DNA virome variations across geography, ethnicity, and urbanization. *Cell Host Microbe* **28**(5), 741–51 e4 (2020). 32910902 10.1016/j.chom.2020.08.005

[45] de Martel, C., Plummer, M., Vignat, J. & Franceschi, S. Worldwide burden of cancer attributable to HPV by site, country and HPV type. *Int. J. Cancer.* **141**(4), 664–70 (2017). 28369882 10.1002/ijc.30716 PMC5520228

[46] Kong, Y. et al. Phylogenetic analysis of Crimean-Congo hemorrhagic fever virus in inner Mongolia, China. *Ticks Tick Borne Dis.* **13**(1), 101856 (2022). 34763306 10.1016/j.ttbdis.2021.101856

[47] Shahhosseini, N. et al. Crimean-Congo hemorrhagic fever virus in Asia, Africa and Europe. *Microorganisms*10.3390/microorganisms9091907 (2021). 34576803 PMC8471816

[48] AlMukdad, S., Harfouche, M., Farooqui, U. S., Aldos, L. & Abu-Raddad, L. J. Epidemiology of herpes simplex virus type 1 and genital herpes in Australia and New Zealand: systematic review, meta-analyses and meta-regressions. *Epidemiol. Infect.* **151**, e33 (2023). 36750224 10.1017/S0950268823000183 PMC9990408

[49] Cunningham, A. L. et al. Prevalence of infection with herpes simplex virus types 1 and 2 in Australia: a nationwide population based survey. *Sex Transm. Infect.* **82**(2), 164–8 (2006). 16581748 10.1136/sti.2005.016899 PMC2564694

[50] Lin, H. et al. Herpes simplex virus infections among rural residents in eastern China. *BMC Infect. Dis.* **11**, 69 (2011). 21414231 10.1186/1471-2334-11-69 PMC3068093

[51] Pirog, A. et al. Comparison of different digestion methods for proteomic analysis of isolated cells and FFPE tissue samples. *Talanta* **233**, 122568 (2021). 34215064 10.1016/j.talanta.2021.122568

[52] Huet, A., Huffman, J. B., Conway, J. F. & Homa, F. L. Role of the herpes simplex virus CVSC proteins at the capsid portal vertex. *J. Virol.* 10.1128/JVI.01534-20 (2020). 32967953 PMC7925205

[53] Le Hars, M., Joussain, C., Jegu, T. & Epstein, A. L. Non-replicative herpes simplex virus genomic and amplicon vectors for gene therapy - an update. *Gene. Ther.* **32**(3), 173–83 (2025). 39533042 10.1038/s41434-024-00500-x PMC12106088

[54] Whitley, R. J. & Roizman, B. Herpes simplex virus infections. *Lancet* **357**(9267), 1513–8 (2001). 11377626 10.1016/S0140-6736(00)04638-9

[55] Tie, Y., Tang, F., Wei, Y. Q. & Wei, X. W. Immunosuppressive cells in cancer: mechanisms and potential therapeutic targets. *J. Hematol. Oncol.* **15**(1), 61 (2022). 35585567 10.1186/s13045-022-01282-8 PMC9118588

[56] Fernandes, Q. & Folorunsho, O. G. Unveiling the nexus: The tumor microenvironment as a strategic frontier in viral cancers. *Cytokine.* **185**, 156827 (2025). 39647395 10.1016/j.cyto.2024.156827

[57] Wang, L. B. et al. Proteogenomic and metabolomic characterization of human glioblastoma. *Cancer Cell* **39**, 509–28 (2021). 33577785 10.1016/j.ccell.2021.01.006 PMC8044053

[58] Zhang, M. et al. ISGylation in innate antiviral immunity and pathogen defense responses: a review. *Front Cell Dev. Biol.* **9**, 788410 (2021). 34901029 10.3389/fcell.2021.788410 PMC8662993

[59] Eaglesham, J. B. & Kranzusch, P. J. Conserved strategies for pathogen evasion of cGAS-STING immunity. *Curr. Opin. Immunol.* **66**, 27–34 (2020). 32339908 10.1016/j.coi.2020.04.002 PMC7158794

[60] Neefjes, J., Jongsma, M. L., Paul, P. & Bakke, O. Towards a systems understanding of MHC class I and MHC class II antigen presentation. *Nat. Rev. Immunol.* **11**(12), 823–36 (2011). 22076556 10.1038/nri3084

[61] Blackford, A. N. & Grand, R. J. Adenovirus E1B 55-kilodalton protein: multiple roles in viral infection and cell transformation. *J. Virol.* **83**(9), 4000–12 (2009). 19211739 10.1128/JVI.02417-08 PMC2668481

[62] Yew, P. R. & Berk, A. J. Inhibition of p53 transactivation required for transformation by adenovirus early 1B protein. *Nature.* **357**(6373), 82–5 (1992). 1533443 10.1038/357082a0

[63] Hartl, B., Zeller, T., Blanchette, P., Kremmer, E. & Dobner, T. Adenovirus type 5 early region 1B 55-kDa oncoprotein can promote cell transformation by a mechanism independent from blocking p53-activated transcription. *Oncogene* **27**(26), 3673–84 (2008). 18212738 10.1038/sj.onc.1211039

[64] Radtke, K., Dohner, K. & Sodeik, B. Viral interactions with the cytoskeleton: a hitchhiker’s guide to the cell. *Cell Microbiol.* **8**(3), 387–400 (2006). 16469052 10.1111/j.1462-5822.2005.00679.x

[65] Leopold, P. L. & Crystal, R. G. Intracellular trafficking of adenovirus: many means to many ends. *Adv. Drug Deliv. Rev.* **59**(8), 810–21 (2007). 17707546 10.1016/j.addr.2007.06.007

[66] Read, J. A., Winter, V. J., Eszes, C. M., Sessions, R. B. & Brady, R. L. Structural basis for altered activity of M- and H-isozyme forms of human lactate dehydrogenase. *Proteins* **43**(2), 175–85 (2001). 11276087 10.1002/1097-0134(20010501)43:2<175::aid-prot1029>3.0.co;2-#

[67] Woodford, M. R. et al. The tumor suppressor folliculin inhibits lactate dehydrogenase A and regulates the warburg effect. *Nat. Struct. Mol. Biol.* **28**(8), 662–70 (2021). 34381247 10.1038/s41594-021-00633-2 PMC9278990

[68] Thai, M. et al. Adenovirus E4ORF1-induced MYC activation promotes host cell anabolic glucose metabolism and virus replication. *Cell Metab.* **19**(4), 694–701 (2014). 24703700 10.1016/j.cmet.2014.03.009 PMC4294542

[69] Prusinkiewicz, M. A. & Mymryk, J. S. Metabolic reprogramming of the host cell by human adenovirus infection. *Viruses* **11**(2), 141 (2019). 30744016 10.3390/v11020141 PMC6409786

[70] Goyal, P. & Rajala, M. S. Reprogramming of glucose metabolism in virus infected cells. *Mol. Cell Biochem.* **478**(11), 2409–18 (2023). 36709223 10.1007/s11010-023-04669-4 PMC9884135

[71] Dybas, J. M. et al. Adenovirus remodeling of the host proteome and host factors associated with viral genomes. *Msystems* **6**, e004621 (2021). 10.1128/msystems.00468-21 PMC12338147 34463575

[72] Liu, Y. et al. KRAB-zinc finger protein ZNF268a deficiency attenuates the virus-induced pro-inflammatory response by Preventing IKK complex assembly. *Cells* **8**(12), 1604 (2019). 31835635 10.3390/cells8121604 PMC6953056

[73] Hu, L. et al. Aberrant expression of ZNF268 alters the growth and migration of ovarian cancer cells. *Oncol. Lett.* **6**(1), 49–54 (2013). 23946776 10.3892/ol.2013.1318 PMC3742507

[74] Wang, W. et al. The zinc finger protein ZNF268 is overexpressed in human cervical cancer and contributes to tumorigenesis via enhancing NF-kappaB signaling. *J. Biol. Chem.* **287**(51), 42856–66 (2012). 23091055 10.1074/jbc.M112.399923 PMC3522282

[75] Weitzman, M. D. & Fradet-Turcotte, A. Virus DNA replication and the Host DNA damage response. *Annu. Rev. Virol.* **5**(1), 141–64 (2018). 29996066 10.1146/annurev-virology-092917-043534 PMC6462412

[76] Locatelli, M. & Faure-Dupuy, S. Virus hijacking of host epigenetic machinery to impair immune response. *J. Virol.* **97**(9), e0065823 (2023). 37656959 10.1128/jvi.00658-23 PMC10537592

[77] Newton, D. A., Rao, K. M., Dluhy, R. A. & Baatz, J. E. Hemoglobin is expressed by alveolar epithelial cells. *J. Biol. Chem.* **281**(9), 5668–76 (2006). 16407281 10.1074/jbc.M509314200

[78] Liu, W., Baker, S. S., Baker, R. D., Nowak, N. J. & Zhu, L. Upregulation of hemoglobin expression by oxidative stress in hepatocytes and its implication in nonalcoholic steatohepatitis. *PLoS One* **6**(9), e24363 (2011). 21931690 10.1371/journal.pone.0024363 PMC3171444

[79] Pagliarini, D. J. et al. A mitochondrial protein compendium elucidates complex I disease biology. *Cell.* **134**(1), 112–23 (2008). 18614015 10.1016/j.cell.2008.06.016 PMC2778844

[80] Foo, J., Bellot, G., Pervaiz, S. & Alonso, S. Mitochondria-mediated oxidative stress during viral infection. *Trends Microbiol.* **30**(7), 679–92 (2022). 35063304 10.1016/j.tim.2021.12.011

[81] Magon, K. L. & Parish, J. L. From infection to cancer: how DNA tumour viruses alter host cell central carbon and lipid metabolism. *Open Biol.* **11**(3), 210004 (2021). 33653084 10.1098/rsob.210004 PMC8061758

[82] Sanchez, E. L. & Lagunoff, M. Viral activation of cellular metabolism. *Virology* **479–480**, 609–18 (2015). 25812764 10.1016/j.virol.2015.02.038 PMC4424078

[83] Mingo-Casas, P. et al. Glycolytic shift during West Nile virus infection provides new therapeutic opportunities. *J. Neuroinflamm.* **20**(1), 217 (2023). 10.1186/s12974-023-02899-3 PMC10537838 37759218

[84] Mansouri, K. et al. Can a metabolism-targeted therapeutic intervention successfully subjugate SARS-COV-2? a scientific rational. *Biomed. Pharmacother.* **131**, 110694 (2020). 32920511 10.1016/j.biopha.2020.110694 PMC7451059

[85] Ganapathy-Kanniappan, S. & Geschwind, J. F. Tumor glycolysis as a target for cancer therapy: progress and prospects. *Mol. Cancer.* **12**, 152 (2013). 24298908 10.1186/1476-4598-12-152 PMC4223729

[86] Munger, J. et al. Systems-level metabolic flux profiling identifies fatty acid synthesis as a target for antiviral therapy. *Nat. Biotechnol.* **26**(10), 1179–86 (2008). 18820684 10.1038/nbt.1500 PMC2825756

[87] Yu, Y., Clippinger, A. J. & Alwine, J. C. Viral effects on metabolism: changes in glucose and glutamine utilization during human cytomegalovirus infection. *Trends Microbiol.* **19**(7), 360–7 (2011). 21570293 10.1016/j.tim.2011.04.002 PMC3130066

[88] Grewal, T., Gerke, V., Nylandsted, J., Rentero, C. & Enrich, C. Revisiting the role of annexins in membrane trafficking. *Cell Mol. Life Sci.* **82**(1), 230 (2025). 40506619 10.1007/s00018-025-05780-z PMC12162462

[89] Taylor, J. R., Skeate, J. G. & Kast, W. M. Annexin A2 in Virus infection. *Front Microbiol.* **9**, 2954 (2018). 30568638 10.3389/fmicb.2018.02954 PMC6290281

[90] Wohl, B. P. & Hearing, P. Role for the L1–52/55K protein in the serotype specificity of adenovirus DNA packaging. *J. Virol.* **82**(10), 5089–92 (2008). 18337584 10.1128/JVI.00040-08 PMC2346739

[91] Lo, A. K., Dawson, C. W., Young, L. S. & Lo, K. W. The role of metabolic reprogramming in gamma-herpesvirus-associated oncogenesis. *Int. J. Cancer.* **141**(8), 1512–21 (2017). 28542909 10.1002/ijc.30795

[92] Mayer, K. A., Stockl, J., Zlabinger, G. J. & Gualdoni, G. A. Hijacking the supplies: metabolism as a novel facet of virus-host interaction. *Front Immunol.* **10**, 1533 (2019). 31333664 10.3389/fimmu.2019.01533 PMC6617997

[93] Suenaga, T. et al. Myelin-associated glycoprotein mediates membrane fusion and entry of neurotropic herpesviruses. *Proc. Natl. Acad. Sci. U S A.* **107**(2), 866–71 (2010). 20080767 10.1073/pnas.0913351107 PMC2818916

[94] Cao, J., Wan, S., Chen, S. & Yang, L. ANXA6: a key molecular player in cancer progression and drug resistance. *Discov. Oncol.* **14**(1), 53 (2023). 37129645 10.1007/s12672-023-00662-x PMC10154440

[95] Mussunoor, S. & Murray, G. I. The role of annexins in tumour development and progression. *J. Pathol.* **216**(2), 131–40 (2008). 18698663 10.1002/path.2400

[96] Grewal, T., Wason, S. J., Enrich, C. & Rentero, C. Annexins - insights from knockout mice. *Biol. Chem.* **397**(10), 1031–53 (2016). 27318360 10.1515/hsz-2016-0168

[97] Ma, H. et al. Human annexin A6 interacts with influenza a virus protein M2 and negatively modulates infection. *J. Virol.* **86**(3), 1789–801 (2012). 22114333 10.1128/JVI.06003-11 PMC3264383

[98] Kulej, K. et al. Time-resolved global and chromatin proteomics during herpes simplex virus type 1 (HSV-1) infection. *Mol. Cell Proteomics.* **16**(4 suppl 1), S92–S107 (2017). 28179408 10.1074/mcp.M116.065987 PMC5393384

[99] Darweesh, M., Mohammadi, S., Rahmati, M., Al-Hamadani, M. & Al-Harrasi, A. Metabolic reprogramming in viral infections: the interplay of glucose metabolism and immune responses. *Front Immunol.* **16**, 1578202 (2025). 40453076 10.3389/fimmu.2025.1578202 PMC12122472

[100] Touat-Hamici, Z., Legrain, Y., Bulteau, A. L. & Chavatte, L. Selective up-regulation of human selenoproteins in response to oxidative stress. *J. Biol. Chem.* **289**(21), 14750–61 (2014). 24706762 10.1074/jbc.M114.551994 PMC4031530

[101] Hannigan, G. E., McDonald, P. C., Walsh, M. P. & Dedhar, S. Integrin-linked kinase: not so ‘pseudo’ after all. *Oncogene* **30**(43), 4375–85 (2011). 21602880 10.1038/onc.2011.177

[102] Guo, W. & Giancotti, F. G. Integrin signalling during tumour progression. *Nat. Rev. Mol. Cell Biol.* **5**(10), 816–26 (2004). 15459662 10.1038/nrm1490

[103] Ong, L. L., Lim, A. P., Er, C. P., Kuznetsov, S. A. & Yu, H. Kinectin-kinesin binding domains and their effects on organelle motility. *J Biol Chem.* **275**(42), 32854–60 (2000). 10913441 10.1074/jbc.M005650200

