# Supplementary material for: Metaproteomic profiling reveals viral proteins and associated host proteomic alterations in glioblastoma
Source: Sci Rep. 2026 Jun 22;16:28566. doi: 10.1038/s41598-026-58378-1 (PMC13572378; doi:10.1038/s41598-026-58378-1)
Supplement: Supplementary file 1 [file 41598_2026_58378_MOESM11_ESM.pdf]

Supplementary File 11: Both spectrums are from the same sample (GB_002)

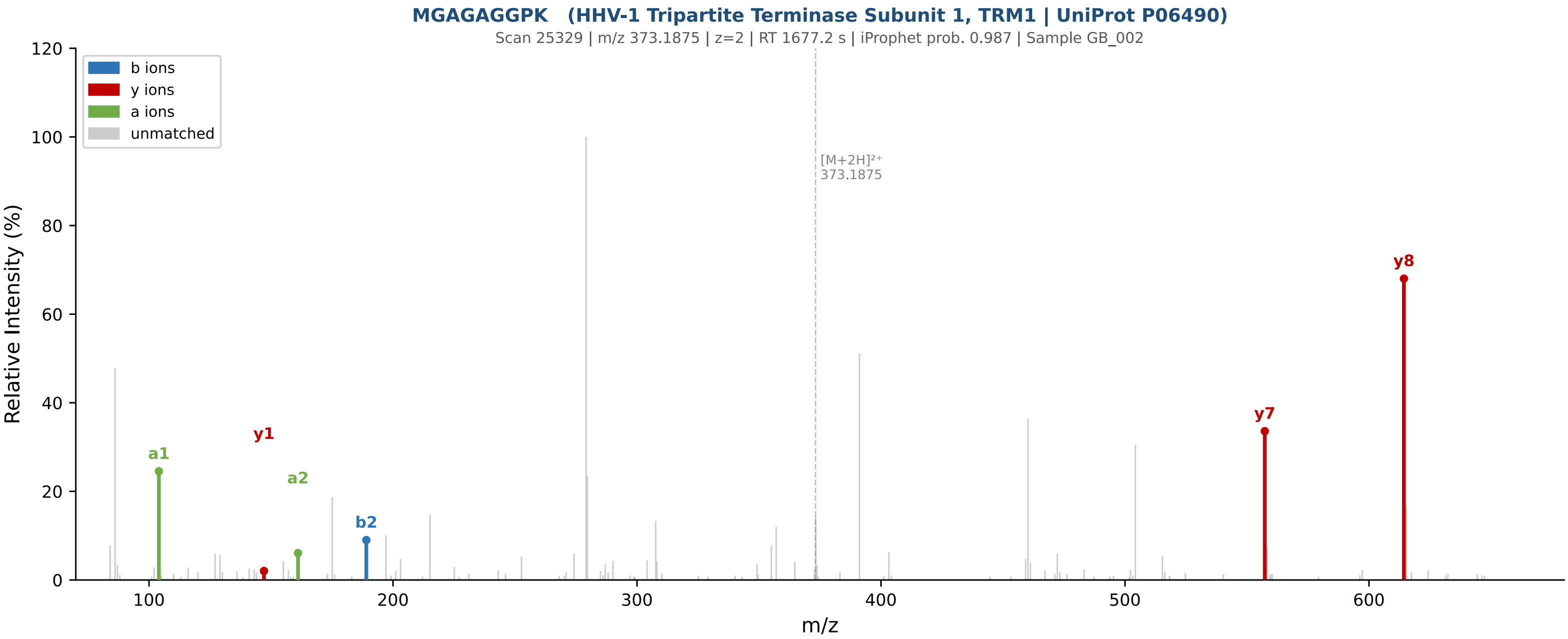

File 11A: MGAGAGGPK- Tripartite terminase subunit 1 (HHV-1) Scan 25329

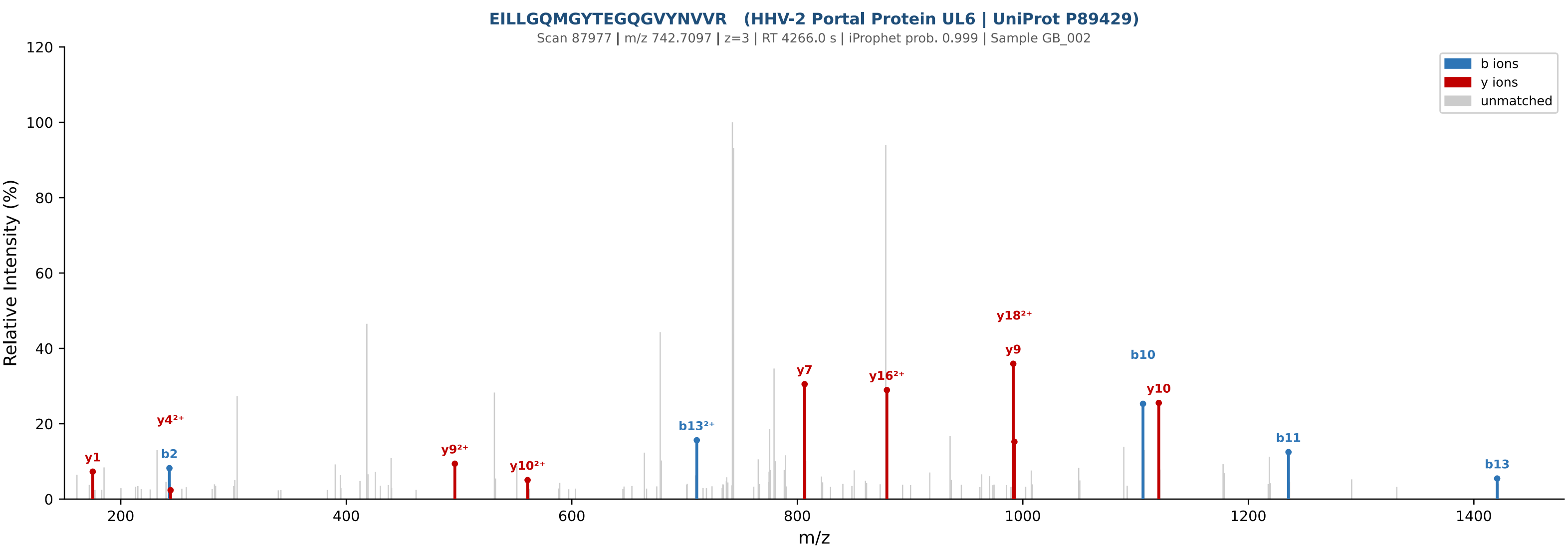

File 11B: EILLGQMGYTEGQGVYNVVR- Portal protein (HHV-2) Scan 87977
