# Supplementary material for: Metaproteomic profiling reveals viral proteins and associated host proteomic alterations in glioblastoma
Source: Sci Rep. 2026 Jun 22;16:28566. doi: 10.1038/s41598-026-58378-1 (PMC13572378; doi:10.1038/s41598-026-58378-1)

Upregulated in tumour

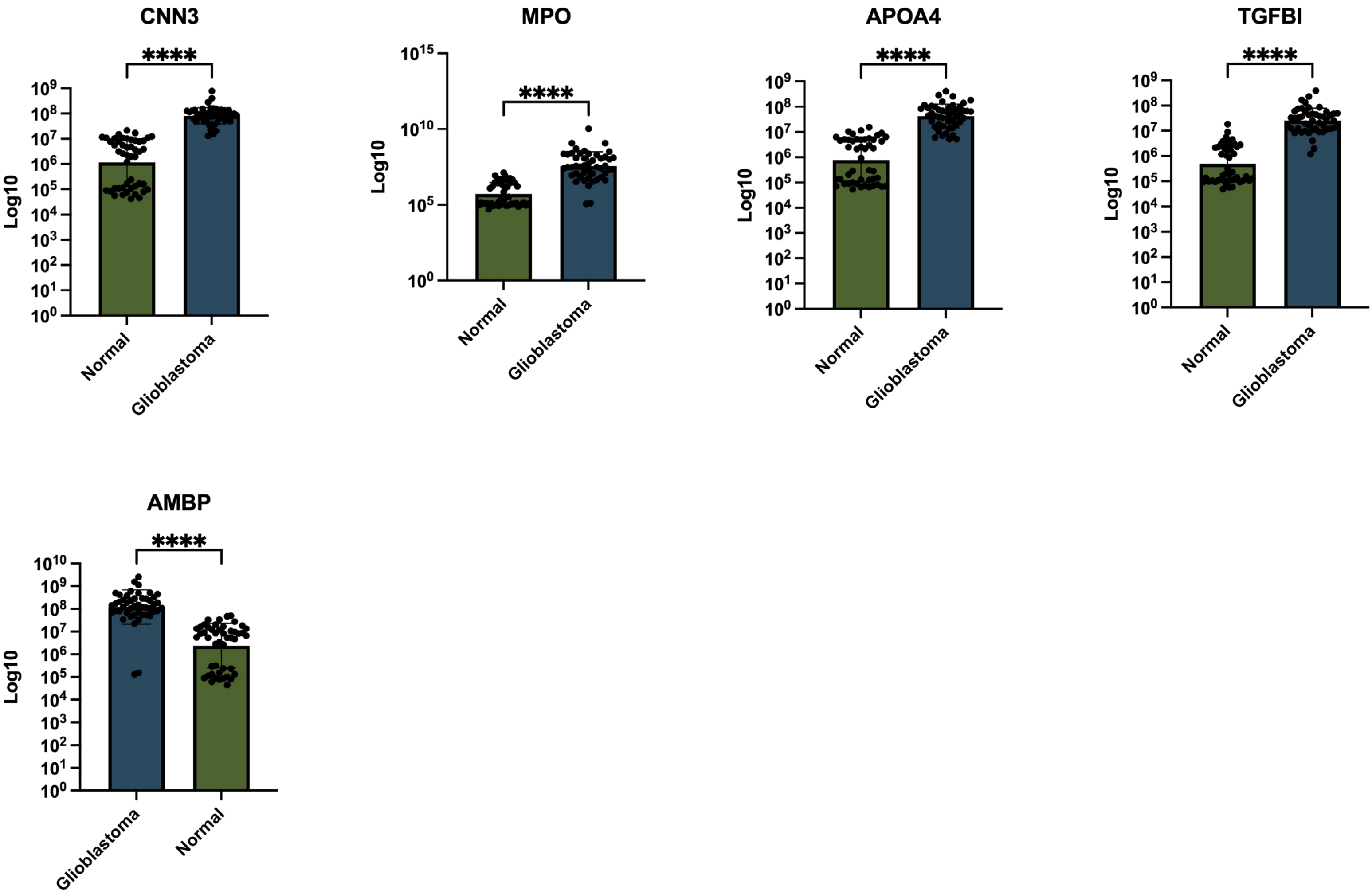

Downregulated in tumour

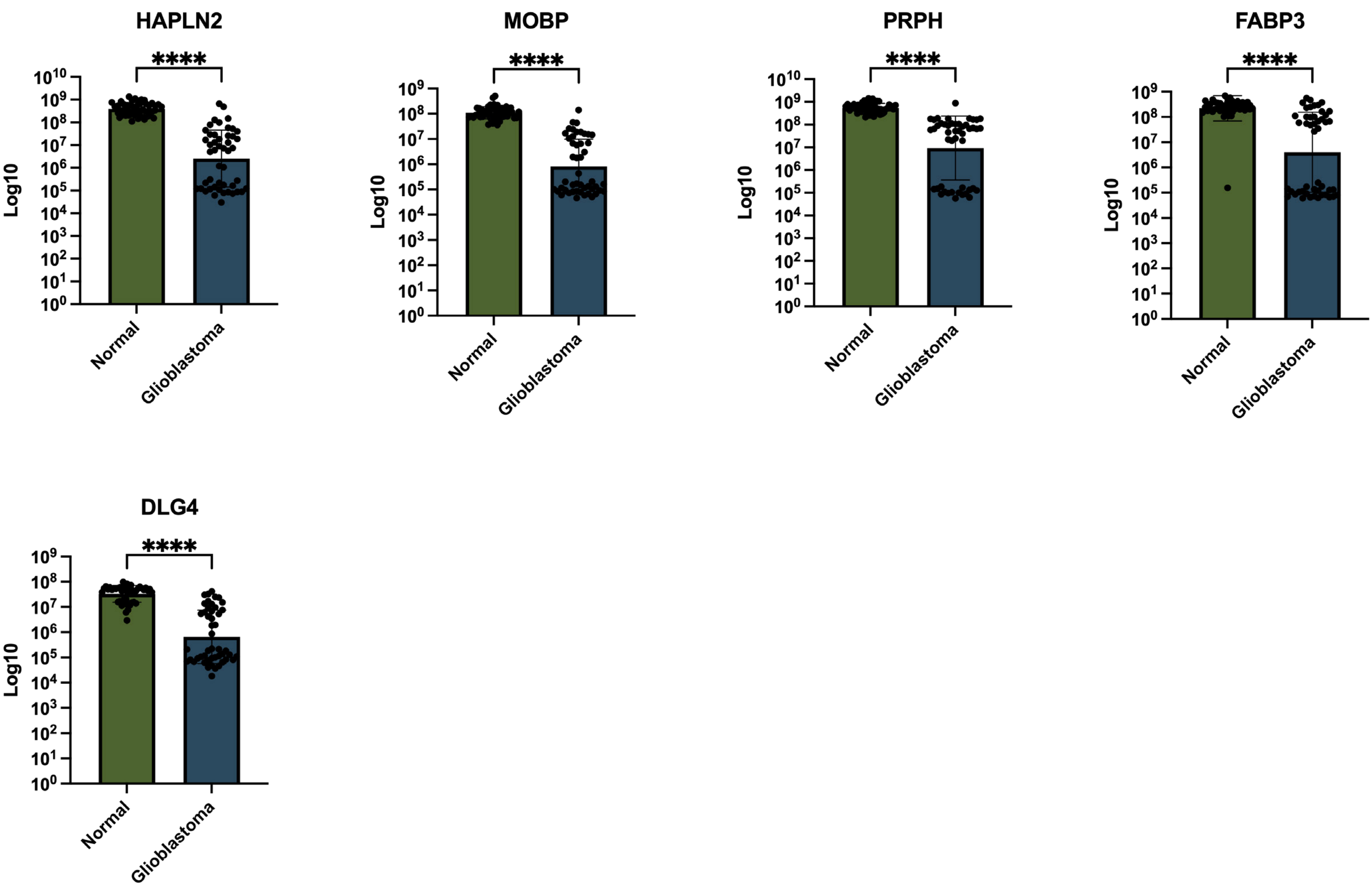

Supplement: Supplementary file 2 [file 41598_2026_58378_MOESM5_ESM.pdf]
